# Integration of O-GlcNAc into Stress Response Pathways

**DOI:** 10.3390/cells11213509

**Published:** 2022-11-05

**Authors:** Kamau M. M. Fahie, Kyriakos N. Papanicolaou, Natasha E. Zachara

**Affiliations:** 1 Department of Biological Chemistry, The Johns Hopkins University School of Medicine, Baltimore, MD 21205, USA; 2 Department of Medicine, Division of Cardiology, The Johns Hopkins University School of Medicine, Baltimore, MD 21205, USA; 3 Department of Oncology, The Johns Hopkins University School of Medicine, Baltimore, MD 21205, USA

**Keywords:** glycosylation, chaperone, stress, O-GlcNAc

## Abstract

The modification of nuclear, mitochondrial, and cytosolic proteins by O-linked βN-acetylglucosamine (O-GlcNAc) has emerged as a dynamic and essential post-translational modification of mammalian proteins. O-GlcNAc is cycled on and off over 5000 proteins in response to diverse stimuli impacting protein function and, in turn, epigenetics and transcription, translation and proteostasis, metabolism, cell structure, and signal transduction. Environmental and physiological injury lead to complex changes in O-GlcNAcylation that impact cell and tissue survival in models of heat shock, osmotic stress, oxidative stress, and hypoxia/reoxygenation injury, as well as ischemic reperfusion injury. Numerous mechanisms that appear to underpin O-GlcNAc-mediated survival include changes in chaperone levels, impacts on the unfolded protein response and integrated stress response, improvements in mitochondrial function, and reduced protein aggregation. Here, we discuss the points at which O-GlcNAc is integrated into the cellular stress response, focusing on the roles it plays in the cardiovascular system and in neurodegeneration.

## 1. Background

Cell fate decisions in response to environmental and physiological stressors are contingent on the integration of multimodal signaling to reprogram/remodel cellular effectors, buffer lethal stimuli, promote survival or engage cell death programs. Stress-responsive remodeling is encoded in part by the covalent modification of proteins. Among the diverse signaling mechanisms directing cellular homeostasis is the addition and removal of O-linked β N-acetylglucosamine (O-GlcNAc) on intracellular proteins, which affects a wide swath of the eukaryotic proteome regulating diverse biological processes, including transcription [1].

O-GlcNAcylation in multicellular eukaryotes is a co-translational and/or post-translational modification (PTM) of nuclear, cytoplasmic, and mitochondrial proteins. With over 5000 human O-GlcNAcylated proteins identified [2,3], O-GlcNAc is implicated in adaptive cellular processes including transcriptional activation and silencing [4,5,6], cell cycle regulation [7], proteostasis [8,9,10], nucleocytoplasmic transport [11], protein–protein interactions [12,13], and signal transduction [14,15,16]. Numerous studies have demonstrated that dynamic protein O-GlcNAcylation is a conserved intracellular signaling mechanism activated by diverse stressors including heat shock [17,18], hydrogen peroxide [17,19], ammonia [20], lipopolysaccharide (LPS) [21] and hypoxia-reoxygenation [22] in cell culture, as well as ischemia-reperfusion (I/R) injury [19], pressure overload [23], and trauma-hemorrhage [24] in animal models. In deciphering the role(s) of O-GlcNAc in the stress response, research depicts general protective effects of raising cellular O-GlcNAc levels before or after an acute stress, while reducing cellular O-GlcNAc levels has deleterious effects [17,19]. Despite general trends demonstrating global increases in O-GlcNAc levels in response to injury, some stressors decrease O-GlcNAc (for instance, trauma-hemorrhage [24]), some display complex dynamics in O-GlcNAc cycling (for instance, oxidative stress in cardiomyocytes [19]), and, in some models, O-GlcNAc levels appear to increase globally, but protein specific analysis revealed nuanced results (for instance, oxidative stress in mouse embryonic fibroblasts (MEFs) [25]). In the latter example, changes in the proteome range from increased O-GlcNAcylation to O-GlcNAc loss [25]. Clearly, decoding O-GlcNAc cycling is key to understanding how it supports homeostatic control. After a brief discussion of the synthesis and cycling of O-GlcNAc, we will explore the impact of regulating global O-GlcNAc levels in different stress models and mechanistic details of O-GlcNAc control of cell stress pathways available in the literature.

## 2. The O-GlcNAc Cycle

**Figure 1:**
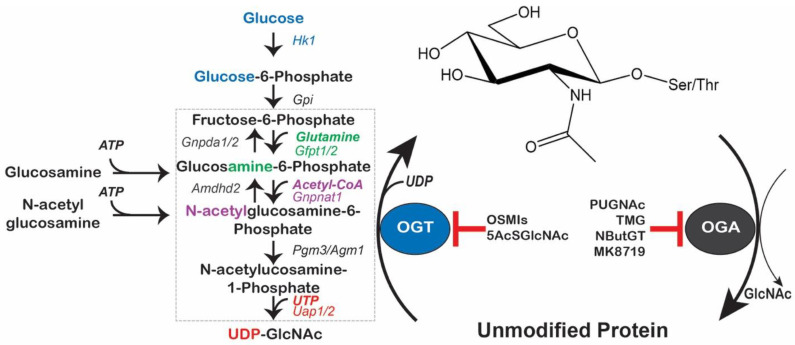
O-GlcNAc cycling. O-GlcNAc is cycled on and off proteins by two enzymes: The O-GlcNAc transferase (OGT) and O-GlcNAcase (OGA), that catalyze the addition and removal of O-GlcNAc, respectively. OGT uses the nucleotide sugar UDP-GlcNAc, which is synthesized by the hexosamine biosynthetic pathways (boxed). Commercially available inhibitors of OGT and OGA are highlighted. Adapted from [1].

The O-GlcNAc cycle is mediated by the “writer” O-GlcNAc transferase (OGT), which uses uridine-5′-diphosphate-N-acetylglucosamine (UDP-GlcNAc) as the sugar donor [1,26], and O-GlcNAcase (OGA), the “eraser” that hydrolyzes the GlcNAc moiety [1,27]. The dynamics of cellular O-GlcNAcylation are dictated by OGT and OGA abundance, enzymatic activity, localization, and substrate targeting/availability, as well as UDP-GlcNAc levels (Figure 1) [1].

### 2.1. Coordination of UDP-GlcNAc Production in Health and Disease

The hexosamine biosynthetic pathway (HBP) branches off from glycolysis (Figure 1, box), utilizing a minor fraction of glucose [28], to generate UDP-GlcNAc in a series of anabolic reactions. UDP-GlcNAc serves as the high-energy sugar donor for a wide spectrum of glycoconjugates, including intracellular protein O-GlcNAcylation. Flux through the HBP is limited by the conversion of fructose-6-phosphate to glucosamine-6-phosphate by the enzyme glutamine:fructose-6 phosphate amidotransferase (GFAT; *Gfpt*). Controlling the first committed step of UDP-GlcNAc synthesis [29,30], the mammalian GFAT paralogs have demonstrated control of global O-GlcNAc levels. For instance, GFAT abundance has been correlated with enhanced O-GlcNAc levels in an inflammation model [21]; macrophages treated with LPS exhibited increased protein O-GlcNAcylation and an increase in GFAT1 abundance. GFAT1/2 is subject to feedback inhibition by UDP-GlcNAc [29,30] and regulation by multiple kinases. 5′AMP-dependent protein kinase (AMPK) [31], calcium/calmodulin-dependent kinase II (CaMKII) [32], and cAMP-dependent protein kinase A (PKA) [33] exhibit different effects on enzymatic activity or expression depending on the identity of the modified residue and the GFAT ortholog.

The ensuing steps in UDP-GlcNAc synthesis involve the conversion of glucosamine-6-phosphate to N-acetylglucosamine-6-phosphate by glucosamine-phosphate N-acetyltransferase (GNPNAT); N-acetylglucosamine-6-phosphate is then isomerized to N-acetylglucosamine-1-phosphate by phosphoglucomutase 3 (PGM3); and UDP–*N*-acetylglucosamine pyrophosphorylase (AGX1, *Uap1*) catalyzes the final step, generating UDP-GlcNAc from N-acetylglucosamine-1-phosphate and UTP. Two enzymes reduce flux through the HBP: glucosamine-6-phosphate deaminase 2 (Gnpda2), which deaminates glucosamine-6-phosphate to fructose-6-phosphate and ammonia [34], and amidohydrolase domain containing 2 (Amdhd2) that deacetylates N-acetylglucosamine-6-phosphate [35]. While less is known about the regulation of these enzymes, recent work suggests that Amdhd2 is a critical controller of HBP flux in tissues where GFAT2 is the predominant GFAT paralog [35]. Flux through the HBP can be manipulated by providing exogenous glucosamine that is imported via the glucose transporter Glut2 or by adding N-acetylglucosamine, which is taken up by endocytosis (Figure 1, left) [36]. Critically, changes in HBP flux may alter cellular function by impacting glycoconjugates independent of O-GlcNAc, including the branching of N-linked glycans [37].

Several lines of evidence suggest that stressors target the HBP. *Firstly,* in a model of the unfolded protein response, elevated UDP-GlcNAc and O-GlcNAc levels were coupled to the enhanced expression and abundance of the HBP enzymes GFAT1, GNPNAT1, and PGM3 [38]. *Secondly*, in tissue culture and *Caenorhabditis elegans* models, increased flux through the HBP has far-reaching physiological effects that include mediating endoplasmic reticulum (ER) stress resistance and longevity [39,40]. *Lastly,* cancer proliferation and progression occur despite the chronic mechanical stress and hypoxia of the tumor microenvironment. Recent studies have pointed to enhanced HBP enzyme expression, notably *Gfpt2* and *Uap1*, as well as enhanced UDP-GlcNAc levels in cancer models and patient samples [41,42]. Downregulating HBP enzyme abundance/activity mitigates cancer cell survival, demonstrating the benefits of HBP rewiring during cancer progression [41].

### 2.2. O-GlcNAc-Cycling by the Writer/Eraser: OGT/OGA

The mammalian OGT is a multi-functional signaling hub catalyzing protein O-GlcNAcylation and proteolysis, as well as having non-catalytic functions [43,44,45]. Knockout studies reveal that mammalian OGT is not only essential for development but critical for post-natal tissue homeostasis. Notably, OGT deletion in the heart and liver leads to cell death and a maladaptive pro-fibrotic response post-injury [46,47,48], suggesting a balancing role for OGT in tissue homeostasis.

OGT is able to regulate a broad range of physiological processes due to its extensive substrate pool [2,3]. Despite lacking a conserved sequon that dictates glycosylation, OGT substrate selection is mediated via contacts spanning the lumen of the N-terminal tetratricopeptide repeat (TPR) domain, with substrates exhibiting differing selectivity among the many TPR motifs [49,50,51]. Substrate selection is also driven by the stable binding partners of OGT [52,53,54]. The glycosyltransferase and proteolytic activity are contained within the same catalytic domain housed in the C-terminus [45]. In responding to different cellular stressors, OGT abundance and/or activity can exhibit transient elevation [17,23,55,56,57,58] that complements the increases in global O-GlcNAc levels. While there are data correlating the covalent modification of OGT to changes in activity, there is insufficient site information to identify regulatory hotspots [59,60,61].

The canonical O-GlcNAcase (OGA) possesses an N-terminal glycosidase domain, an intervening stalk domain, and a C-terminal histone acetyltransferase (HAT)-like domain [62,63,64,65,66]. Like OGT, the canonical OGA exhibits nuclear and cytoplasmic localization, whereas an isoform lacking the HAT-like domain exhibits mitochondrial and lipid droplet association [67,68,69]. OGA is subject to cleavage by caspase 3 during apoptosis [70,71], and high-throughput proteomic studies have identified PTMs on the canonical OGA [72,73,74], though the effects of these PTMs on activity or substrate specificity are yet to be determined. OGA is also stress-responsive [56,58]. While global changes in O-GlcNAc levels can exhibit temporal profiles with stress, increasing and decreasing with time, activity assays demonstrate that OGA activity can be enhanced despite global enhancement of O-GlcNAc [52,55]. Consistent with these phenomena, proteomic data suggest that O-GlcNAc removal occurs on some proteins despite the global increase [25].

Several mechanisms are thought to regulate the expression and abundance of OGT and OGA. MicroRNAs (miRs) have been reported to target both OGT (miR122) and OGA (mIR539) [75,76]. O-GlcNAc levels also control the maturation of OGT and OGA mRNA by regulation of detained intron splicing [77,78]. Here, elevated levels of O-GlcNAc inhibit OGT mRNA maturation and promote OGA maturation, resulting in enhanced OGA and reduced OGT abundance. Conversely, low levels of O-GlcNAc promote maturation of OGT mRNA and inhibit that of OGA [77,78]. Although the mechanisms of stress responsive changes in OGT and OGA protein abundance are currently undefined, detained intron splicing is a potential mechanism controlling these phenomena, as seen with Clk1/4 stress-induced intron splicing [79]. In the heart, OGA splicing is impacted by protein arginine methyltransferase 5 (PRMT5) [80], whose absence from cardiomyocytes leads to cardiac dilation, fibrosis, and age-related lethality. Consequently, *Prmt5* deficiency leads to decreased abundance of OGA and increased overall O-GlcNAcylation. Changes in the maturation of OGA mRNA are also detected in human dilated cardiomyopathy, demonstrating the importance of maintaining OGA expression for normal cardiac function [80].

While there are thousands of O-GlcNAcylated substrates, there are only two enzymes that add and remove O-GlcNAc. Residues in both OGT and OGA beyond their respective active sites provide substrate targeting [45,50,81]; however, it is broadly accepted that protein interactors also play a role in targeting OGT and OGA to substrates. The potential of these protein interactors to mediate biology is highlighted by a recent study identifying protein interactors impacted by pathologic mutations in OGT [82]. Recently, two studies have addressed changes in OGT and OGA interactors induced by cellular stress [52,55]. A BioID proximity labeling approach was employed to reveal the stress-regulated (H_2_O_2_/2 h) interactome of OGA in U2OS cells [55]. These studies identified 85 high-confidence OGA interactors, of which 21 exhibited a greater than 25% increase in their interaction with OGA upon stress. Further interrogation confirmed the stress-induced interaction of OGA with fatty acid synthase (FASN), filamin A (FLNA) and heat shock cognate 70 (Hsc70). Notably, the association of OGA with FAS was inhibitory to its catalytic activity. These data suggest that protein interactors may inhibit OGA activity during stress, leading to enhanced O-GlcNAcylation of a subset of proteins [55]. Such data may underpin the nuanced changes in O-GlcNAcylation highlighted by proteomics experiments [25].

In a complementary study, the stress-induced interactome of OGT was investigated [52]. OGT was enriched from mouse embryonic fibroblasts (MEFs) exposed to oxidative stress (H_2_O_2_/1.5 h). Subsequently, proteins were identified and their association with OGT quantified by SILAC proteomics [52]. This analysis revealed 47 baseline interactors of OGT and 119 stress-induced interactors. The interactors identified did not fall into a specific functional category but rather encompassed diverse protein classes, including carboxylic acid metabolism, redox reactions, and glucose and fatty acid metabolism [52]. The screen identified well-known OGT interactors/substrates such as host cell factor 1 (HCF1) and protein arginine methyl transferase 4 (PRMT4/Carm1), as well as novel interactors such as Bag6, a cochaperone involved in protein quality control, and tankyrase 1 binding protein (Tnks1bp1) involved in DNA double strand break repair and actin filament dynamics. The diverse spectrum of stress interactors suggests that OGT localizes in various macromolecular complexes within the cell, enabling broad and rapid effects in response to stress [52].

## 3. Impact of O-GlcNAc in Physiological Models of Injury and Pathophysiology

**Figure 2:**
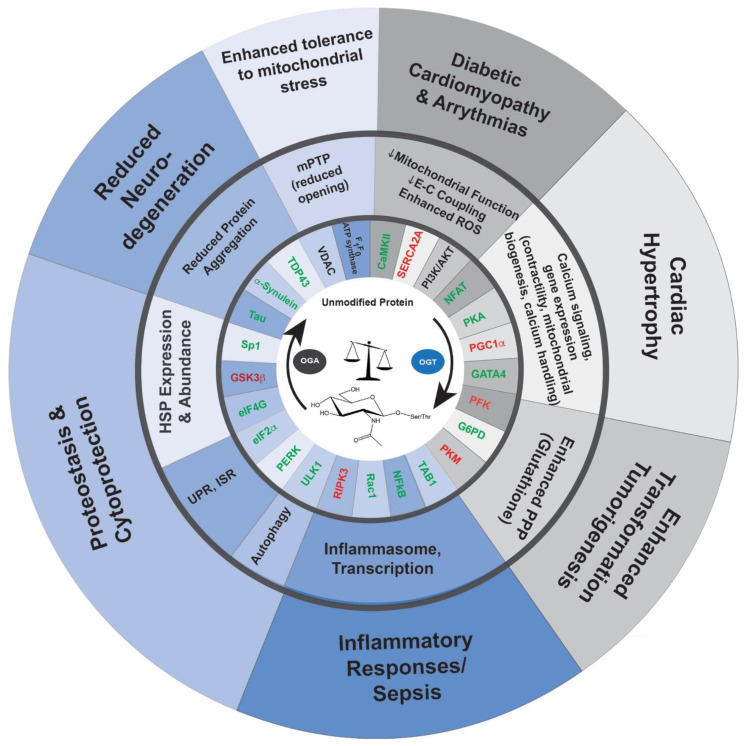
Mechanisms of Protection and Pathogenesis. Dynamic cycling of O-GlcNAc plays numerous roles in regulating cellular homeostasis and the response to cellular injury (blue). In contrast, chronic elevation or depletion of O-GlcNAc resulting in aberrant O-GlcNAc cycling is associated with disease pathogenesis (grey), including cardiomyopathy. Proteins whose function is potentiated by O-GlcNAc are highlighted in green, whereas those whose function is inhibited are highlighted in red. Glycoproteins impacted by O-GlcNAc through ill-defined mechanisms are identified in black. Abbreviations used in this figure: **AKT:** Protein kinase B; **CaMKII:** Calcium/calmodulin-dependent protein kinase II; **E-C:** Excitation-contraction; **eIF2α:** Eukaryotic translation initiation factor 2-Alpha; **eIF4G1:** Eukaryotic translation initiation factor 4 Gamma, 1; **ER:** Endoplasmic reticulum; **G6PD:** Glucose-6-phosphate dehydrogenase; **Gata4:** GATA binding protein 4; **GSK3β**—Glycogen Synthase Kinase β; **HSP:** Heat shock protein; **IKK:** I-kappa B kinase complex; **ISR,** Integrated Stress Response; **mPTP:** Mitochondrial permeability transition pore; **NF-κB:** Nuclear factor kappa B subunit 1; **NFAT:** Nuclear factor of activated T cells; **O-GlcNAc:** O-linked b N-acetylglucosamine; **OGA:** O-GlcNAcase; **OGT:** O-GlcNAc transferase **PERK:** PKR-like ER kinase; **PFK1:** Phosphofructokinase 1; **PGC1α:** Peroxisome Proliferative Activated Receptor, Gamma, Coactivator 1, Alpha; **PGK1:** Phosphoglycerate kinase 1; **PI3K:** Phosphatidylinositol-3-kinase; **PKA:** cAMP-dependent protein kinase; **PKM2:** Pyruvate kinase splice isoform 2; **PPP,** Pentose Phosphate Pathway; **Rac1:** Ras-related C3 botulinum toxin substrate 1; **RIPK:** Receptor-interacting serine/threonine kinase; **ROS:** Reactive oxygen species; **SERCA2A:** Sarcoplasmic/endoplasmic reticulum calcium ATPase 2; **TAB1:** TAK1-binding protein 1; **TDP43:** Transactive response DNA binding protein 43; **ULK1:** Unc-51 like autophagy activating kinase 1; **VDAC:** Voltage dependent anion channel.

Changes in O-GlcNAc-cycling have been implicated in a host of disease pathologies, including neurodegenerative disease, cardiomyopathy, numerous forms of cancer, X-linked disability, and metabolic syndrome [1]. Below, we discuss those pathologies in which the stress response plays a critical role (Figure 2).

### 3.1. Cardiac Development, Physiology, Pathophysiology, and Homeostasis

Mice with germline deletion of OGT are not viable due to impaired stem cell viability during embryogenesis [83,84]. Conditional deletion of OGT specifically in cardiomyocytes is compatible with embryonic survival, but the knockout mice have impaired post-natal survival and develop dilated cardiomyopathy [47]. OGT-deficient mice surviving to adulthood have fibrosis, cardiomyocyte hypertrophy, and elevated markers of ER stress [47]. It is likely that the impaired postnatal survival is due in part to cardiac developmental defects occurring in utero that result in hyper-trabeculation, septal defects, and incomplete development of coronary vessels [85]. These defects appear to be associated with decreased cardiomyocyte proliferation and poor angiopoietin expression but are independent of hypoxia-inducible factor-1α (Hif1α) and NK2 transcription factor related locus 5 (Nkx2-5) [85]. While these findings suggest that loss of O-GlcNAcylation impairs the development of cardiomyocytes, numerous studies suggest that enhanced O-GlcNAcylation for prolonged periods of time also disrupts cell and tissue function. For example, abnormally high levels of O-GlcNAcylation can delay the differentiation of embryonic stem (ES) cells into cardiomyocytes [86]. Overexpression experiments (OGT/GFAT) in HEK293 cells identified an inverse relationship between O-GlcNAcylation and protein levels of the master regulator of cardiogenesis—Nkx2-5 [87] OGT and Nkx2-5 were found to interact in both HEK293 cells and mouse hearts [86,87]; however, O-GlcNAcylation was not detected on Nkx2-5 isolated from cardiac tissue [85]. Other models [88,89,90], including cardiac mesenchymal stromal cells [91], also display an inverse relationship between O-GlcNAcylation and stem cell differentiation [18,19,20].

Mice with transgenic overexpression of OGT (cmOGT-Tg) under the myosin heavy chain 6 (*Myh6*) promoter (active in cardiomyocytes from middle embryonic to postnatal development and in adulthood) do not exhibit any overt post-natal developmental defects and lethality but exhibit an early decline in heart function at 6 weeks of age [92]. A key driver of pathology in the cmOGT-Tg mice appears to be impaired activity of mitochondrial Complex I. Importantly, interbreeding cmOGT-Tg with a mouse line expressing OGA under the *Myh6* promoter (cmOGA-Tg) normalizes O-GlcNAcylation levels and prevents Complex I impairment, suggesting that excess O-GlcNAcylation is detrimental to Complex I activity [92]. Interestingly, proteomic profiling in cardiac mitochondria identified numerous subunits of Complex I as targets of O-GlcNAcylation, and acutely elevating O-GlcNAcylation with Thiamet-G (TMG; OGA inhibitor) boosts mitochondrial energetics [93]. Similarly, short-term elevation of O-GlcNAcylation by exposing mitochondria to 1,2-dideoxy-2′-propyl-α-d-glucopyranoso-[2,1-d]-Δ2′-thiazoline (NButGT; OGA inhibitor) increases O-GlcNAcylation on respiratory complexes, including Complex I, and enhances its activity and overall mitochondrial respiration [94]. Therefore, short-term increases in Complex I O-GlcNAcylation appear beneficial to its function, but like stem cell differentiation, long-term hyper O-GlcNAcylation is detrimental.

Mice with inducible adult-onset OGT cardiomyocyte deletion (I-cmOGT KO) develop progressive cardiomyopathy that becomes apparent after the first month of tamoxifen treatment [47]. Investigation at 5 or 10 days post-OGT deletion found that knockout mice did not have signs of overt cardiac dysfunction but exhibited increased expression of Glyceraldehyde-3-phosphate dehydrogenase (*Gapdh)* and alterations in autophagy markers [46,95]. Furthermore, I-cmOGT KO cardiomyocytes examined 18–30 days post deletion had decreased intracellular Ca^2+^ release and contractility, signifying aberrant excitation–contraction (E–C) coupling [96]. These phenotypes may result from downregulation of Ca_v_ α1 and β2 subunits of the voltage-dependent L-type Ca^2+^ channel [96]. Interestingly, these I-cmOGT KO hearts also exhibit increased overall protein ubiquitination and neddylation [96]. The intersection between O-GlcNAc and Ca^2+^ signaling has been previously noted in cardiac myocytes, where, for example, O-GlcNAcylation affects the aberrant activation of CaMKII [97,98] and the regulation of store-operated calcium entry via STIM1/Orai1 channels [99,100].

Genetic inactivation of OGA results in perinatal lethality that is associated with reduced intrauterine growth, inability to thrive, lung defects, depletion of the liver glycogen stores, and low levels of circulating glucose [101,102]. Similarly, mice with catalytically inactive OGA (D285A) show small embryo size and perinatal lethality accompanied by structural defects in embryonic kidney, liver, and brain [103]. While no major defects in the cardiovascular system were detected in OGA D285A mice, depleting OGA by 50% in adult cardiomyocytes (OGA^+/−^-icko) elevates cardiac O-GlcNAcylation and modestly reduces ejection fraction at baseline [104]. These data suggest that OGA has important maintenance roles in the heart and cardiomyocytes. In contrast to these data, overexpression of OGA in cardiomyocytes (cmOGA-Tg) results in reduced O-GlcNAcylation and slight cardiac hypertrophy that is not accompanied by enhanced expression of *natriuretic peptide alpha* (*Nppa*) and *myosin heavy chain 7* (*Myh7*; markers of pathologic hypertrophy) [92]. Consistently, the cmOGA-Tg heart does not exhibit any significant functional defects at baseline [92]. Collectively, it appears that enhancing the expression of OGA in cardiomyocytes does not impact the heart function significantly, possibly as O-GlcNAc levels can be controlled by proteolysis of target proteins and OGT activity/abundance (*as discussed above*).

Diabetes disrupts O-GlcNAcylation in cardiac mitochondria [105], where several important foci, including oxidative/phosphorylation subunits, enzymes of the Krebs cycle and fatty acid oxidation, exhibit widespread changes in their O-GlcNAcylation [93]. Early experiments noted that decreasing O-GlcNAcylation by overexpressing OGA in cardiomyocytes ameliorates mitochondrial dysfunction caused by exposure to high glucose and found that this is associated with improved function of respiratory complexes [106]. Viral delivery of OGA in induced pluripotent stem cell (iPSC)-derived cardiomyocytes limited the mitochondrial dysfunction caused by high glucose [107]. Notably, viral delivery of OGA to the heart protected against the development of diabetic cardiomyopathy, and this was correlated with decreased cardiac hyper-O-GlcNAcylation of phosphatidylinositol-3-kinase (PI3K), protein kinase B (AKT), and sarco/endoplasmic reticulum Ca2+-ATPase (SERCA2A) [107]. In addition to restoring mitochondrial function and PI3K-dependent signaling, OGA was found to improve the contractility of myofilaments in diabetic hearts by removing excess O-GlcNAcylation [108].

The Ca^2+^/calmodulin-dependent kinase CaMKII is a master regulator of acute stress responses in the heart that phosphorylates and regulates diverse classes of ion channels in cardiac myocytes. O-GlcNAcylation of CaMKII at Ser280 renders it overactive in the context of diabetes or hyperglycemia [97,109]. Acute exposure of cardiac myocytes to high glucose (30 mM) induces the production of reactive oxygen species (ROS) and sarcoplasmic reticulum (SR) Ca^2+^ leaks that can be prevented by inhibition of CaMKII with KN93, inhibition of OGT (with OSMI-1), or mutation of CaMKII into the O-GlcNAc-resistant Ser280Ala [109]. These effects are consistent with a model where O-GlcNAcylation at S280 renders CaMKII overactive, which in turn then increases ROS production via NADPH oxidase and SR Ca^2+^ leak via phosphorylation of the ryanodine receptor (RyR). Similarly, cardiomyocytes from diabetic animals exhibit pro-arrhythmogenic action potentials when exposed to high glucose that are abolished in cardiomyocytes derived from CaMKII Ser280Ala hearts [98]. Ser280 O-GlcNAcylation and overactivation of CaMKII are also implicated in the aberrant function of sarcolemmal potassium (K^+^) channels [98]. In this model, overactive CaMKII impacts the abundance, phosphorylation and activity of sarcolemmal K+ channels, decreasing the net K+ current in diabetic cardiomyocytes. Consistent with the previous observations, the aberrant regulation of K^+^ channels is prevented in the O-GlcNAcylation-resistant CaMKII Ser280Ala mutant cardiomyocytes. Intriguingly, a separate line of investigations examining CaMKII’s role in diabetes-induced susceptibility to atrial fibrillation found no significant protection in mice harboring the Ser280Ala mutation [110]. While this study found that excess O-GlcNAcylation in the context of diabetes plays a key role in atrial fibrillation, it highlights that targets other than CaMKII Ser280 are implicated in this pathogenic mechanism.

### 3.2. Pressure Overload Hypertrophy, Myocardial Infarction, and Ischemia Reperfusion Models

In myocardial biopsies of patients with aortic stenosis, O-GlcNAcylation was found to be elevated, as was the abundance of OGT and OGA, suggesting that O-GlcNAc may play a role in hypertrophy [111]. The model of pressure overload hypertrophy in mice, or rats, involves a combination of mechanical and neurohumoral stresses leading to an initially adaptive growth of the heart and cardiomyocytes, due to active protein synthesis. Following the early phase, sustained pressure overload induces maladaptive cardiac remodeling characterized by myocyte death, fibrosis, ventricle dilation and declining contractility, all hallmarks of heart failure. Several studies identified increased protein O-GlcNAcylation due to pressure overload (PO) [23,111,112,113,114]. However, PO-dependent O-GlcNAcylation appears to undergo dynamic regulation, with an increase in the early phase (1 week) and a decrease to baseline levels in the late phase (6 weeks) [23]. Consistent with a protective role of O-GlcNAcylation in hypertrophy, I-cmOGT KO hearts subjected to PO exhibit contractile dysfunction [113,115]. Furthermore, deleting OGT after establishing hypertrophy (18 days of PO) potently exacerbates dysfunction, indicating protective roles of O-GlcNAcylation not just in early hypertrophic response but also in cardiac remodeling [115]. In contrast, administering TMG (OGA inhibitor), did not significantly ameliorate the cardiac hypertrophy, and if anything, it elevated the stress marker *Nppa* [23]. Finally, I-cmOGA-Tg mice exhibiting reduced myocardial O-GlcNAcylation were protected from PO hypertrophic stress [92]. Therefore, the levels of O-GlcNAcylation in the heart are not always predictive of PO-induced cardiac remodeling, and other parameters such as the stage of hypertrophy (early or established), the duration (short- or long-term), and magnitude of O-GlcNAc manipulation are key contributors to the final outcome.

Several studies have investigated whether altering the HBP impacts PO hypertrophy. GFAT is subject to regulation by AMPK [116]. In this model, AMPK phosphorylates and inhibits GFAT to limit cardiac O-GlcNAcylation, and this is sufficient to ameliorate hypertrophy due to PO or isoproterenol (ISO) infusion stress [116]. GFAT is present in two isoforms; while GFAT 1 is widely expressed, GFAT 2 is expressed selectively in the cardiac fibroblasts [117]. Overexpression of GFAT 1 in cardiac myocytes exacerbates PO hypertrophy, while its deletion from cardiomyocytes is protective against hypertrophy and contractile dysfunction [118]. In addition to GFAT 1, emerging evidence shows that GFAT 2 is upregulated in hearts subject to ISO stress. Furthermore, concomitant infusion of ISO with 5-diazo-oxo-norleucine (DON; GFAT inhibitor) ameliorates the stress-induced hypertrophy together with reduced O-GlcNAcylation levels [119]. Thus, the evidence currently indicates that targeting the GFAT/UDP-GlcNAc signaling axis can be beneficial by preventing maladaptive effects of chronic increased O-GlcNAcylation in the stressed heart. Mechanistically, candidate O-GlcNAc targets impacting the outcome of PO hypertrophy include the catalytic subunit of PKA and its regulation of phospholamban (a key calcium-handling protein in the heart) [28], as well as the transcription factors nuclear factor of activated T-cells (NFAT), GATA Binding Protein 4 (GATA4), Peroxisome proliferator-activated receptor gamma coactivator-1 alpha (PGC1α), and X-box binding protein 1 spliced (Xbp1s) [23,112,113,120]. These molecular targets thus implicate O-GlcNAcylation in diverse cardiac stress response pathways, including calcineurin and calcium handling, adaptive gene expression of contractile and metabolic genes, mitochondrial biogenesis, and the unfolded protein response (UPR).

In addition to PO hypertrophy cardiac stress, O-GlcNAcylation has also been investigated in cardiac remodeling following myocardial infarction (MI). Failing human hearts biopsied at the time of transplantation exhibit increased O-GlcNAcylation [104]. Similarly, investigations in animal models with acute MI and heart failure found increased cardiac O-GlcNAcylation, accompanied by increased OGT and decreased OGA abundance or activities [46,121]. Consistent with a protective role of O-GlcNAcylation, loss of cardiomyocyte OGT leads to detrimental cardiac remodeling and heart failure following acute MI [46]. These findings indicate that boosting O-GlcNAcylation exerts a protective role in the context of MI and heart failure. Nevertheless, mice with partial deletion of OGA in cardiomyocytes, while exhibiting increased myocardial O-GlcNAcylation, unexpectedly undergo exacerbated myocardial remodeling and dysfunction after MI [104]. These apparently conflicting outcomes are reminiscent of the findings with pressure overload hypertrophy described above. There are several potential explanations: non-catalytic roles of OGT and OGA or the prolonged perturbation of O-GlcNAc-cycling per se (by inhibiting one of the two cycling enzymes), rather than the net increase or decrease in O-GlcNAcylation, is the detrimental element that exacerbates cardiac dysfunction during stress.

Myocardial stress resulting from transient I/R injury is one of the first in vivo stress models that was found to be impacted by O-GlcNAcylation [19]. Initial work focused on ex vivo cardiac perfusion experiments in which O-GlcNAc levels were found to first increase during early time points of ischemia, but then to decline at later time points in ischemia and during reperfusion [122,123]. Maintaining high O-GlcNAcylation levels in reperfusion, by glucosamine supplementation or OGA inhibition (Figure 1), was shown to be protective against ex vivo I/R injury [122,124,125,126]. These data were recapitulated in vivo by injecting mice with the OGA inhibitor PUGNAc prior to MI, resulting in reduced infarct size [19]. Increased O-GlcNAcylation was observed in models of acute, prolonged, and remote ischemic preconditioning, indicating that O-GlcNAcylation may underpin endogenous cardioprotective pathways [19,56,58].

Opening of the mitochondrial permeability transition pore (mPTP) underlies many of the pathological outcomes of I/R injury. Consistently, loss of OGT in cardiomyocytes exacerbates mPTP opening and cell damage [19,127], while enhancing O-GlcNAcylation (TMG, 18 h) prevents mPTP opening [91]. Similarly, enhancing O-GlcNAcylation (I.P. injection TMG) in the heart prevents mPTP opening [94]. The mitochondrial protein voltage-dependent anion channel (VDAC), a potential subunit of the mPTP, has been identified as O-GlcNAc-modified in the heart [19]. Another potential subunit of the mPTP, the F_1_F_o_ ATP synthase [128] is frequently found in cardiac/mitochondrial O-GlcNAcomes [69,94].

### 3.3. ER Stress Response

The ER stress response, which arises from the accumulation of misfolded proteins in the ER or ER-calcium depletion, may also mediate cellular responses in an O-GlcNAc-dependent manner. Early reports in Cos-7 cells and cardiomyocytes found that ER stress (Tunicamycin, dithiothreitol) augments O-GlcNAcylation [17,129], and boosting O-GlcNAcylation with OGT overexpression or OGA inhibition reduced expression of ER stress markers [129]. In vivo, elevated levels of O-GlcNAc in response to ER stress initiated by myocardial I/R injury are partly attributed to the ER stress signal transducer Xbp1s [38]. Xbp1s is induced early in reperfusion (30 min) and acts as a transcription factor that promotes the expression of enzymes within the HBP, raising GFAT1 and UDP-GlcNAc levels at 24 h post-MI [38]. In adipocytes, a key mediator of ER stress, the ER-resident protein kinase (PERK), was shown to interact with and promote the phosphorylation of OGT, resulting in the stimulation O-GlcNAcylation of downstream targets at ER-mitochondrial contact sites [130].

O-GlcNAcylation may directly regulate protein translation, and thus impact ER-stress responses, by modifying ribosomal proteins and eukaryotic translation initiation factors (eIF) [131,132]. For example, O-GlcNAcylation was identified on translation initiation factor eIF2α, a key regulator of translation initiation rates [133]. Several studies have demonstrated that the induction of heat shock proteins (HSPs) is regulated by O-GlcNAc, with enhanced levels of O-GlcNAc associated with faster induction of HSPs [17,18,134]. One mechanism underpinning this phenotype is O-GlcNAcylation of factor eIF4G1 at its N-terminus (Ser68) [135]. In this model, eIF4G1 interacts with eIF4E and polyA binding protein 1 (PABP1) to form stalled pre-initiation complexes containing the small ribosomal subunit and the *Hsp70* mRNA at normal temperatures. However, upon heat stress, eIF4G1 is O-GlcNAcylated and its interaction with eIF4E is relaxed, allowing activation of the pre-initiation complex and selective translation of *Hsp70* mRNA [135].

Cardiomyocyte-specific deficiency of *Ogt* is associated with upregulation of ER stress markers 78 KDa Glucose Regulated Protein (GRP78/BiP) and protein disulfide isomerase (PDI) [47]. Similarly, *Ogt* deficiency in pancreatic β cells is accompanied by induction of ER stress markers including CCAAT/enhancer-binding protein homologous protein (CHOP) and GRP78 [136]. Haploinsufficiency of *Chop* in the context of pancreatic *Ogt* deficiency is protective against injury and the development of glucose intolerance [136]. Further investigation in pancreatic β cells found that the translation initiation scaffold protein eIF4G1 undergoes O-GlcNAcylation (Ser61), and this is important for its stability and downstream expression of pancreatic carboxypeptidase E (CPE), a key insulin-processing enzyme [134].

While loss of O-GlcNAcylation appears to contribute to the induction of ER stress that can be deleterious to cellular function, there is also evidence that excess O-GlcNAcylation is a trigger of ER stress. For example, glucose deprivation significantly elevates O-GlcNAcylation in cardiac myocytes and induces ER stress markers including increased phosphorylation of PERK and accumulation of GRP78 and CHOP proteins [137]. In that setting, activation of ER stress appears to be downstream of increased intracellular Ca^2+^ and activation of the calmodulin-dependent phosphatase calcineurin [137]. Nevertheless, many details remain unknown on how glucose deprivation stimulates O-GlcNAcylation and whether this is a cause or a correlate of ER stress. In other cell experiments, glucose deprivation induces the expression of OGT [138], an effect that is mediated by AMPK, and promotes the interaction of p38 MAP kinase with OGT that targets the latter to substrates [54]. The translational repressor eIF4E binding protein 1 (4E-BP1) undergoes O-GlcNAcylation in diabetic retinopathy, and this promotes its stabilization and interaction with eIF4E [139]. Enhanced eIF4E/4E-BP1 interaction strongly suppresses the translation of mRNAs that require a 7-methyl guanosine cap structure for their translation and favors cap-independent translation [140]. Consistently, enhancement of O-GlcNAcylation in the retina by OGA inhibition (TMG) globally alters the patterns of mRNA translation, leading to suppression of mitochondrial proteins and enhanced production of mitochondrial ROS [141].

### 3.4. Trauma Hemorrhage

Despite advances in emergency medicine, traumatic hemorrhage (TH) resulting from traffic accidents or conventional warfare remains a leading cause of death. Severe injury and hemorrhagic shock can induce hyperglycemia, which is thought to be an adaptive mechanism [140,141]. Based on the observation that flux through the HBP increased in response to hyperglycemia, the role of O-GlcNAc in trauma hemorrhage has been examined. Early studies found that protein O-GlcNAcylation remained unchanged in the heart and brain in a rat model (90 min hemorrhage, 60 min fluid resuscitation, 2 h recovery) [142]. Nevertheless, increasing O-GlcNAcylation by glucosamine treatment or OGA inhibition (PUGNAc) was associated with improved cardiac function and peripheral organ perfusion with [24,142] or without fluid resuscitation [143]. The protective effects of glucosamine were associated with reduced inflammatory markers including tumor necrosis factor-alpha (*Tnfa*) and Interleukin 6 (*IL6*) mRNAs, NF-kappa-B inhibitor alpha (IκBα) phosphorylation, and nuclear localization of nuclear factor NF-kappa-B (NF-κB) [144]. Mechanistic information on how O-GlcNAcylation attenuates NF-κB signaling in the TH heart remains elusive. Interestingly, existing evidence suggests that NF-κB is activated directly by O-GlcNAcylation [145,146]. Further studies investigating the effect of metabolic manipulation (glucosamine) and OGA inhibition (PUGNAc) on survival found that while both treatments had a significant impact on survival 24 h after hemorrhagic shock (from 53% to 85% and 86%, respectively), only OGA inhibition protected the heart as well as the liver and the lungs [147]. Further investigation is necessary to fully elucidate how acutely elevating O-GlcNAcylation mitigates the proinflammatory responses activated by trauma hemorrhage in the various tissues.

In septic shock models (endotoxemia induced by LPS injection or cecal ligature and puncture), the treatment with the OGA inhibitor NButGT (3 or 24 h after shock induction) increased O-GlcNAc levels in the heart and ameliorated pathologic parameters including depressed ejection fraction, shock-induced tachycardia, and the release of creatinine, as well as improving survival [148]. The expression of inflammatory markers (*IL6*, *Tnfa*) in the heart was not significantly impacted by NButGT, although NButGT significantly prevented a shock-induced increase in the abundance of SERCA2A. Follow-up studies using proteomic approaches found several proteins exhibiting differential O-GlcNAcylation upon septic shock and treatment with NButGT [149]. Among these were heat shock proteins (HSP70, HSP27), mitochondrial respiratory subunits, metabolic enzymes (Protein disulfide-isomerase A4 and ATP-citrate synthase), and components of the contractile apparatus and cytoskeleton (Tubulin alpha, Troponin C, Lamin A) [149]. Finally, a transcriptomic analysis of this model revealed differential expression of genes participating in mitogen-activated protein kinase (MAPK), NF-κB and Janus kinase-signal transducer and activator of transcription (JAK-STAT) signaling pathways [150]. However, while these pathways were strongly impacted by LPS endotoxemic stress, they were not affected by NButGT treatment. One explanation is that the short window of OGA inhibition (2 h, NButGT) does not sufficiently capture early O-GlcNAc-related changes in gene expression [150]. Together, these studies highlight a beneficial role of O-GlcNAcylation in septic shock and imply that its beneficial effects in the early phase are likely attributable to direct O-GlcNAcylation on diverse cardiac proteins across multiple different functional classes.

### 3.5. O-GlcNAc Cycling Resists Proteotoxic Aggregation in Neurodegenerative Diseases

Proteostasis dysfunction is one of the commonalities of neurodegenerative diseases that reflects an imbalance in the networks controlling synthesis, folding, trafficking, and degradation of the proteome. While the affected proteins are disease-specific, the formation of protein aggregates/inclusion bodies in different compartments typifies the proteotoxic insult, altering biomolecular interactions, and sequestering/mislocalizing cellular proteins, causing an imbalance in cellular processes [151,152]. Prime examples include the trapping of chaperones and proteasome components in inclusions [153]. Defects in protein quality control and mitochondrial function exacerbate the deleterious effects of the inclusion bodies, eventually leading to cell death.

#### 3.5.1. Alzheimer’s Disease

Collapse of proteome stability in Alzheimer’s disease (AD) is typified by the extra- and intraneuronal accumulation of amyloid-β (Aβ) plaques and intracellular Tau inclusions, also known as the neurofibrillary tangle (NFT). Premature neurodegeneration in a conditional deletion of *Ogt* in the adult mouse forebrain displayed pathogenic processing of Tau and amyloid precursor protein (APP) despite lacking a disease-prone genetic background [154]. This observation suggests that O-GlcNAc signaling functions as a general safeguard for proteome stability. Further evidence supporting a role for O-GlcNAc in buffering proteotoxicity was seen in patient samples where global O-GlcNAc levels and/or OGT expression were suppressed relative to control samples [155,156]. Quantitative proteomic data suggest that the impact of AD on O-GlcNAc is more nuanced, indicating altered, reduced, and enhanced, O-GlcNAcylation of proteins in AD patients. The altered proteins in question belong to diverse classes including synaptic structural proteins and regulatory mRNA binding proteins [157,158]. These data suggest that O-GlcNAc likely mitigates neurodegeneration through multiple pathways; however, to date, the focus of most functional studies has been on Tau and APP.

Following the initial observation of Tau O-GlcNAc modification in vitro [159], analysis of AD patient samples revealed a significant reduction in global O-GlcNAc levels and a decrease in Tau O-GlcNAcylation [157,160]. Site-mapping of in vitro O-GlcNAcylated recombinant Tau identified multiple sites including Ser400, which was observed in mouse and rat lysate using a site-specific Tau Ser400 O-GlcNAc antibody [161,162]. In vitro aggregation studies provide insight regarding how direct O-GlcNAcylation resists Tau derangement. The direct O-GlcNAc modification of Tau significantly lowered its aggregation propensity, without adversely affecting its canonical functions [162,163,164]. In vivo evidence also supports a role for O-GlcNAcylated Tau resisting aggregation. In mouse models, elevating O-GlcNAc levels suppressed Tau NFT formation, neuronal loss, and neurobehavioral decline. This is exemplified in studies employing OGA inhibitors: TMG and MK-8719 [162,165,166,167].

The role of O-GlcNAc in regulating Tau proteostasis takes on more dimensions when considering the complex interplay of the Tau PTMs, including phosphorylation, acetylation, methylation, and ubiquitination in directing Tau fate [168]. Hyperphosphorylation of Tau is a signature of AD progression. Elevating O-GlcNAc by inhibiting OGA (TMG) in PC-12 cells and healthy mouse and rat brains not only raised Tau O-GlcNAcylation but also reduced Tau phosphorylation at multiple sites [157,169]. Suppressing Tau O-GlcNAcylation via starvation in mice increased Tau phosphorylation [157,160]. Collectively, these data suggested that O-GlcNAc may suppress aggregation by negatively impacting phosphorylation. However, a reduction in Tau phosphorylation in response to raising cellular O-GlcNAc levels has not been universally reported: while two studies observed reduced phosphorylation of Tau in response to OGA inhibition (TMG, MK-8719) in rTg4510 mice [166,170], phosphorylation was not markedly affected in the JNPL3 and Tau.P301L AD mouse models (TMG) [162,165]. The inverse relationship between Tau O-GlcNAcylation and phosphorylation in the aforementioned studies may reflect PTM interplay that is more relevant to normal physiology, as the observations were made in the absence of the AD pathological factors. Further efforts will be needed to ascertain the information encoded by physiological Tau O-GlcNAcylation given the diverse interactors Tau encounters [171,172].

The tauopathy animal studies present multiple possibilities regarding O-GlcNAc suppression of Tau aggregation. The inverse trends reflected in reducing insoluble Tau while increasing O-GlcNAc-modified Tau may be the direct result of enhanced conformational stability as observed in the in vitro models. Given the fast aggregation kinetics of hyperphosphorylated recombinant Tau [173], the inconsistencies in the in vivo crosstalk of Tau phosphorylation and O-GlcNAc suggest that other O-GlcNAc-regulated pathways may participate in suppressing aggregation. Macroautophagy (autophagy) and chaperone-mediated autophagy (CMA) both function in Tau clearance (soluble and aggregates) [174,175] with autophagy-related genes (ATGs) and other regulatory proteins subject to O-GlcNAc cycling [176,177].

In addition to Tau, β-amyloid deposition in animal AD models was also responsive to global changes in neuronal O-GlcNAc levels. The cellular fate of APP is determined by many layers of proteostasis, including trafficking, intramembrane proteolysis, and lysosomal degradation. Widely expressed in the brain, APP is a transmembrane glycoprotein that functions in neurite outgrowth, synaptogenesis, and plasticity [178]. A type-1 membrane protein, the APP N-terminus is extra-cellular, and the short C-terminus is cytosolic. Physiological and pathophysiological APP processing entails proteolysis at different subcellular locations: two sites in the extracellular domain catalyzed by either α- or β-secretase and intramembrane cleavage by γ-secretase. The reputed non-amyloidogenic processing involves cleavage by α-secretase liberating a soluble fragment (sAPPα), followed by cleavage of the membrane-bound fragment by γ-secretase generating the extracellular p3 peptide, disfavoring amyloid-β peptide (Aβ) formation. It should be noted that there is debate regarding the amyloidogenic potential of the p3 peptide that aggregates in vitro and in patient plaques [179,180]. The “classic” amyloidogenic processing involves sequential proteolytic events by β- and γ-secretase producing soluble APPβ (sAPPβ) and the well-studied Aβ deposited in AD patients. APP trafficking between the plasma membrane, the trans-Golgi network, and endosomes modulates cleavage events; α-secretase proteolysis occurs preferentially at the cell membrane, while endocytic trafficking favors Aβ generation. Raising cellular O-GlcNAc levels with OGA inhibitors (NButGT, TMG, and PUGNAc) in the AD-susceptible 5XFAD mice and tau/APP mice enhanced cognitive performance, reduced Aβ peptide levels, and suppressed amyloid plaque deposition [181,182]. Underlying these observations, γ-secretase, crucial for extracellular Aβ production, displayed lower activity in brain lysates of 5XFAD mice treated with NButGT [182]. The proposed regulatory mechanisms suppressing amyloid deposition include suppressing γ-secretase activity via direct O-GlcNAcylation of the nicastrin subunit at Ser708 [182], increased non-amyloidogenic processing of APP as indicated by higher sAPPα secretion [183], and suppressed endocytosis of amyloidogenic peptides associated with APP O-GlcNAcylation [184,185,186]. Though the end results of reduced amyloid plaque deposition and neuronal death were observed across numerous studies, divergence in the proposed protective mechanisms may reflect the differences in the model systems utilized for the studies, as well as the influence of other O-GlcNAc-regulated pathways activated by OGA suppression.

#### 3.5.2. α-Synuclein O-GlcNAcylation Suppresses Aggregation and Prion-like Behavior That Typifies Parkinson’s Disease

Highly enriched in presynaptic terminals, α-synuclein regulates vesicle fusion and neurotransmitter release in normal physiology [187]. Current research indicates that physiological α-synuclein is subject to dynamic equilibrium between an unstructured monomer and an α-helical multimer [188,189,190], with multimerization implicated in binding the synaptic vesicle membrane. Misfolded α-synuclein oligomers accumulate in neuropathology, causing progressive synaptic dysfunction, membrane permeabilization, prion-like cell-to-cell spreading, and eventual neuronal death. The insoluble intracellular Lewy bodies formed by misfolded α-synuclein oligomers are one of the proteotoxic aggregates that are characteristic of advanced-stage Parkinson’s disease. The pathogenic α-synuclein has been directly linked to diverse gene modifications (missense mutations and gene multiplication) and some PTMs [191,192]. Unlike the disease-associated PTMs, O-GlcNAcylation represents a subset of the protective PTMs identified.

Multiple O-GlcNAc sites on α-synuclein have been identified in mice and humans, including Thr72, Thr75, Thr81 and Ser87 [193]. Elevating global O-GlcNAcylation (TMG) has been demonstrated to enhance α-synuclein abundance in neurons, pointing to the potential of O-GlcNAc to promote neuronal death [194]. However, the majority of data from studies of site-specific O-GlcNAcylation suggest that O-GlcNAc suppresses α-synuclein aggregation and toxicity. In vitro aggregation studies utilizing synthetic α-synuclein demonstrated that the homogeneous O-GlcNAcylation of Thr72 suppresses aggregation of homogenously modified α-synuclein, without affecting α-helical multimerization upon membrane binding [195,196]. In addition, modification of α-synuclein Thr72 with O-GlcNAc slowed aggregation in heterogenous mixtures with low percentages of modified synuclein. Homogeneous O-GlcNAcylation of Ser87 also hindered aggregation without negatively affecting membrane binding, though with lower effectiveness compared to Thr72 O-GlcNAcylation [196,197]. Analyses of the aggregation propensity of α-synuclein homogenously O-GlcNAc-modified at the other known sites (Thr75, Thr81) revealed that the O-GlcNAc modification generally hindered aggregation in vitro, with site-specific differences in aggregation kinetics. Notably, modification at Thr81 displayed the most inhibitory effect [196].

In addition to suppressing formation of misfolded oligomers, O-GlcNAc cycling was shown to restrict the prion-like behavior of α-synuclein oligomers [195,198]. The latter was demonstrated using different approaches: (1) The cytotoxicity of aggregates generated from unmodified or O-GlcNAcylated (Thr72) α-synuclein was assessed in neuronal culture; (2) unmodified α-synuclein fibril uptake was measured in neuroblastoma culture in which cellular O-GlcNAc levels were up- or downregulated. The exogenous addition of O-GlcNAcylated α-synuclein aggregates did not produce significant toxicity compared to the unmodified α-synuclein aggregates [195]. Fibril uptake of unmodified α-synuclein was restricted when cellular O-GlcNAc levels were upregulated via OGA inhibition or knockdown, while downregulating O-GlcNAc levels, via OGT inhibition, enhanced fibril uptake [198]. The neuroprotective effects garnered via α-synuclein O-GlcNAcylation or enhanced cellular O-GlcNAc levels suggest that the direct modification makes α-synuclein a less suitable endocytic cargo and raising O-GlcNAc levels blunts the endocytic route utilized by α-synuclein oligomers. OGA inhibition did not impede endocytosis of other cargo, including cholera toxin and HIV-1 Tat [198]. Towards uncovering how enhancing cellular O-GlcNAc blocks the endocytic route, chemical synthetic methodology may be highly advantageous. Cell surface proteins have been implicated in α-synuclein fibril internalization [199,200,201]; as such, the inclusion of photo-reactive crosslinking residues on the exposed surface of α-synuclein fibrils could help identify the molecular interactions that promote internalization and are disrupted by raising cellular O-GlcNAc levels.

#### 3.5.3. O-GlcNAc Modification of Transactive Response DNA Binding Protein 43 Resists Aggregation Observed in Amyotrophic Lateral Sclerosis

Amyotrophic lateral sclerosis (ALS) is a neurodegenerative disease characterized by progressive motor deficits due to motor neuron loss. Initially classified for the presentation of motor dysfunction, clinical manifestations in up to half of patients highlight a multisystem degeneration with more than 20 genes implicated in ALS [202,203]. Cytoplasmic proteotoxic insults are a common signature of ALS with several different aggregating proteins identified, including superoxide dismutase 1 (SOD1), fused in sarcoma (FUS), and transactive response DNA binding protein 43 (TDP-43), a focal point in the study of disease pathogenesis. TDP-43′s physiological roles encompass RNA stability, splicing, transcription, and gene regulation. Liquid–liquid phase separation of physiological TDP-43 has been linked to RNA binding, whereas pathological TDP-43 forms gel-like to solid inclusions with nonfunctional sequestration of ubiquitin and proteasome components and other aberrant biomolecular interactions [153,204].

Given the role of PTMs in modulating biomolecular condensates and protein aggregation, research into TDP-43 has identified covalent modifications affecting functional activity, nucleocytoplasmic distribution [205] and phase transitions [206,207]. A recent report from Zhao et al. [208] demonstrated the O-GlcNAcylation of endogenous TDP-43 in SH-SY5Y cells. TDP-43 O-GlcNAcylation was responsive to alterations in global O-GlcNAcylation via overexpression of wildtype and catalytically inactive OGT and OGA or treatment with OGT and OGA inhibitors (OSMI-1 and TMG). Enhancing TDP-43 O-GlcNAcylation suppressed aggregation in yeast and mammalian cells. Mutations of TDP-43 at Thr199 and Thr233 compromised TDP-43 O-GlcNAcylation, reduced alternative RNA splicing capacity, and partially rescued locomotor defects in *Drosophila melanogaster* relative to wildtype TDP-43 overexpression. New questions arise regarding pathogenesis—Do ALS features reflect the toxic biomolecular interactions due to TDP-43 aggregation, or is impaired RNA processing the more significant contributor?

Studies indicate that ROS accumulation in ALS [209] is a contributing factor in TDP-43 aggregation [210,211]; therefore, suppression of ROS accumulation offers another method of ameliorating TDP-43 aggregation observed in both sporadic and familial ALS [212,213]. Deletion of the nonselenocysteine-containing phospholipid hydroperoxide glutathione peroxidase (NPGPx or GPx7) in mice phenocopies the locomotor deficits and motor neuron degeneration characteristic of ALS [211]. The NPGPx/GPx7 knockout mice also exhibit a dramatic reduction in global O-GlcNAc levels consistent with the reduced O-GlcNAc levels in the mutant SOD1 ALS mouse model [214]. Raising cellular O-GlcNAc levels via OGA inhibition (TMG) suppressed motor neuron loss, and locomotion deficits were improved in the NPGPx/GPx7 knockout mice [215]. These data strongly indicate that aberrant O-GlcNAc cycling contributes to the ALS phenotype. OGA inhibition (TMG) reduced ROS levels and cell death in cells depleted of NPGPx/GPx7, implicating O-GlcNAc signaling in downregulating ROS accumulation [215]. Given that ROS is a contributing factor in TDP-43 aggregation, O-GlcNAc signaling may confer TDP-43 conformational stability by downregulating cellular ROS levels in addition to stabilization via TDP-43 O-GlcNAc modification.

## 4. Other Stress-Responsive Pathways Regulated by O-GlcNAc

The advent of high-sensitivity mass spectrometers, the development of fragmentation techniques that preserve the glycan attachment sites, and the optimization of appropriate enrichment strategies have led to many publications describing the O-GlcNAcome in cells and tissues basally or after exposure to physiological or pathological stimuli. As a result of the growing information, recent efforts have organized the O-GlcNAcome (target proteins and sites) in the form of searchable databases [2,3]. These databases suggest that more than 5000 human proteins are modified by O-GlcNAc, and that these proteins fall into functional classes including RNA metabolism, cellular responses to external stimuli, amino acid and protein metabolism, cell cycle regulation, development, and signal transduction [3].

One mechanism for identifying proteins that underpin the cytoprotective role that O-GlcNAc plays is to identify proteins whose O-GlcNAcylation is cycled in response to injury. At least four studies have addressed this goal, using antibodies that recognize terminal βGlcNAc residues (CTD110.6) [216], antibodies that demonstrate high specificity for O-GlcNAc and variable dependency on the peptide backbone [217], or metabolic labeling of glycans in cells with clickable unnatural O-GlcNAc analogs [218]. These approaches have been applied to models that include trauma hemorrhage (rat liver) [217], oxidative stress in MEFs (H_2_O_2_; 1 and 2 h) [25], heat shock in Cos-7 cells [219], and Adriamycin genotoxic stress in human breast cancer cells. These studies suggest that hundreds of proteins are temporally targeted by both OGT and OGA. Several themes have emerged, including identification of proteins from pathways regulating protein folding (HSPs), chromatin regulators, transcription regulation, vesicle transport (Cop II vesicle transport proteins), nuclear pore proteins, metabolic enzymes, methyltransferases, oxidative stress (SOD1, Thioredoxin and Gsta2), RNA biogenesis, and DNA damage-dependent protein kinase [25,217,218,219]. These data support the hypothesis that O-GlcNAc cycling in response to stress remodels cellular pathways impacting survival. The impact of O-GlcNAc on a subset of proteins has been discussed above with respect to disease pathogenesis; below, we address the role of O-GlcNAc in the regulation of pathways critical to responding to injury.

### 4.1. O-GlcNAc Cycling Mediates Pro- and Anti-Inflammatory Signaling

#### 4.1.1. The Role of O-GlcNAcylation in the Activation and Resolution of the Inflammatory Response

An integral component of physiology, inflammation is a response to perturbations meant to restore tissue homeostasis. The classic inflammatory response is triggered by environmental factors, including traumatic damage, exogenous molecules, and invading pathogens; however, genetic susceptibility and metabolic derangement are also contributing factors seen in chronic inflammation [220,221]. The acute inflammatory response is characterized by the induction of chemokine and cytokine expression, vasodilation and increased vascular permeability, granulocyte recruitment to the site of inflammation, neutralization and removal of pathogenic agents, and ultimately the resolution or termination of the inflammatory response [222]. The intercellular chemokine–cytokine communication network is key to directing immune cell and non-immune cell-activity for pro- and anti-inflammatory outcomes.

Several studies have detailed the involvement of protein O-GlcNAcylation in mediating the pro-inflammatory signaling cascades at multiple points. For instance, the activation of Transforming growth factor β-activated kinase 1 (TAK1), a pro-inflammatory transducer, requires O-GlcNAcylation of a cognate binding partner for full activation [15]. TAK1 activation is regulated by various pro-inflammatory agents, including interleukin-2 (IL-2), transforming growth factor-β (TGF-β), TNFα, LPS, the Toll-like receptors, CD40 and B cell receptor (BCR) [223,224]. These pro-inflammatory molecules mediate TAK1 activation via autophosphorylation, which then phosphorylates several targets in the inflammatory signaling cascade: mitogen-activated kinase kinases (MKK) 3/6, MKK4/7, and the I-kappa B kinase (IKK). This signaling cascade culminates in the upregulation of inflammatory genes under the control of the transcription factors AP-1 and NFκB [225,226].

In addition to autophosphorylation, TAK1 activation is also regulated by the O-GlcNAcylation of its binding partner TAK1-binding protein 1 (TAB1) [15]. The functional TAK1 complex consists of the constitutively bound regulatory subunit TAB1 and context-dependent association of TAB2 or TAB3. TAB4 enhances TAK1 autophosphorylation and forms a stable complex with TAK1 and the other TABs in neutrophils. The covalent modification of TAB1-3 has differential effects on TAK1 activity, adding multiple layers of complexity to TAK1 regulation [223]. A recent study showed that TAB1 undergoes O-GlcNAcylation in vivo in response to hyperglycemia as well as IL-1 and osmotic stress, demonstrating that TAB1 O-GlcNAcylation is controlled by activators of TAK1 signaling [15]. The TAB1 O-GlcNAcylation site was unequivocally identified as Ser395, using mass spectrometry and mutational analyses. More significantly, the TAB1 Ser395Ala mutant revealed a significant reduction in TAK1 kinase activity, IκBα phosphorylation, NFκB transcriptional activity, and IL-6 and TNFα secretion compared to wildtype cells [15]. Clearly, TAB1 O-GlcNAcylation is required for full TAK1 activation, NFκB activation and cytokine secretion. Immunoprecipitated endogenous TAB3 from MDA-MB-231 cells was O-GlcNAcylated [227], and proteomic datasets point to O-GlcNAcylation of TAB2 [228].

Pro-inflammatory events downstream of TAK1 activation also utilize O-GlcNAc signaling as a positive regulatory mechanism. Yang and co-workers (2008) have highlighted the importance of direct O-GlcNAcylation of RelA/p65 in upregulating the transcriptional activity of the NFκB complex in rat vascular smooth muscle cells (VSMCs). Conditions enhancing cellular O-GlcNAc levels, including hyperglycemia and OGA inhibition (PUGNAc), increased NFκB O-GlcNAcylation and transcriptional activity [145,229]. Towards delineating the mechanism of O-GlcNAc-dependent NFκB activation, Yang et al. (2008) demonstrated that the O-GlcNAcylation of p65/NFκB destabilizes its interaction with the inhibitor of nuclear factor kappa B (IκB) protein in the cytosol. Disruption of this interaction unmasks the NFκB nuclear localization signal and culminates in increased nuclear translocation of p65, as seen in the scenario of hyperglycemia-induced NFκB activation. Mutation of a major p65 O-GlcNAc site, Ser352, lowered the nuclear localization and transcriptional activity of NFκB. O-GlcNAcylation of the c-Rel subunit at Ser350 was also shown to promote transcriptional activity [229].

Disruption of the inhibitory NFκB-IκB complex is also achieved via O-GlcNAcylation of IKK, a pro-inflammatory regulator [230]. IKK specifically phosphorylates IκB, promoting the dissociation of IκB from NFκB and the degradation of the IκB protein. IKKβ O-GlcNAcylation was induced under high glucose conditions in MEFs, human fibroblasts and NIH 3T3 cells. Enhanced IKKβ O-GlcNAcylation corresponded to increased phosphorylation of Ser181 in the IKK activation loop. Interestingly, mutation of Ser733 (S733A, S733E) significantly abates IKK O-GlcNAcylation. As Ser733 is a reported inhibitory phosphorylation site for IKK [231,232], it likely participates in direct or indirect PTM crosstalk with IKK O-GlcNAcylation. Either Ser733 is phosphorylated or O-GlcNAc-modified in a mutually exclusive fashion, or Ser733 is needed for recognition of a proximal O-GlcNAc site that was disrupted by the point mutation in the study.

Neutrophils are recruited to sites of inflammation, moving towards high chemoattractant concentrations. The bacterial chemoattractant N-formyl-methionine-leucine-phenylalanine (fMLP) rapidly enhanced polymorphonuclear leukocyte (PMN) protein O-GlcNAcylation within 1 min of stimulation, with O-GlcNAc levels returning to resting levels after 10 min [233]. fMLP-induced O-GlcNAc signaling highlights the responsiveness of O-GlcNAc-cycling enzymes to this G-protein coupled receptor (GPCR)-mediated pathway. Furthermore, enhancing UDP-GlcNAc levels and global protein O-GlcNAcylation (glucosamine) increased basal and fMLP-induced chemotaxis [233]. Subsequently, the authors identified elements of chemotactic signaling that displayed upregulated activity in response to enhanced cellular O-GlcNAcylation. During neutrophil chemotaxis, the Rac1 guanosine triphosphatase (GTPase) is key to direction sensing. Indeed, Rac1 was rapidly activated following fMLP stimulation in neutrophils [234]. Elevating the O-GlcNAc level using metabolic manipulation (glucosamine) and OGA inhibition (PUGNAc) augmented Rac1 activity under basal and fMLP stimulation. Raising cellular O-GlcNAc levels also enhanced activation of Rac1 downstream effectors, p38 MAPK and p44/42 MAPK, under basal and fMLP induction. At present, it is unclear which components of the Rac1 motility signaling pathway undergo O-GlcNAc cycling during chemotaxis, though OGT was shown to interact with p38 MAPK in other biochemical contexts [54]. Further experimentation on Rac1 motility signaling may yield definitive O-GlcNAc-modified targets regulating chemotaxis.

Intriguing molecular details pertaining to the role of O-GlcNAcylation in inflammation resolution have been discovered. A prime example is the effect of receptor-interacting serine/threonine kinase 3 (RIPK3) O-GlcNAcylation in suppressing inflammation and necroptosis in myeloid-derived cells [235]. The NLRP3 inflammasome functions in pro-inflammatory cytokine maturation, and RIPK3 is a key activator of the inflammasome [236,237]. Septic inflammation and mortality were heightened in mice engineered with myeloid-specific *Ogt* deletion. OGT inhibition (OSMI-1) in bone marrow-derived macrophages (BMMs) from wildtype mice also displayed heightened inflammation with significant LPS-triggered cytokine production [238]. The hyperinflammation cytokine profile was mirrored in the *Ogt*-deficient BMMs, further validating the role of OGT function in suppressing inflammasome activation [235]. RIPK3 activation was notably upregulated in the *Ogt*-deficient BMMs, and loss of RIPK3 suppressed the hyperinflammation profile of OGT-deficient BMMs. The authors demonstrated that RIPK3 was O-GlcNAc-modified in wildtype BMMs but not in the *Ogt*-deficient BMMs. Mass spectrometry identified several potential O-GlcNAcylation sites, including Thr467. Consequently, mutation of Thr467 enhanced RIPK3 activation, downstream signaling and cytokine expression with LPS stimulation [235].

As early as 1980, several reports indicated that increased HBP flux has anti-inflammatory potential, as metabolic manipulation with glucosamine and N-acetylglucosamine exerted a suppressive effect on inflammation. In models of osteoarthritis, glucosamine has been shown to reduce the expression of Cox-2, a source of pro-inflammatory prostaglandins, IL-2, and IL-6 [239,240]. Another hallmark of inflammation is the expression of matrix metalloproteinases (MMPs), especially MMP-2, -3, -9 and -12, which are not only induced in response to inflammation activation but are also implicated in chemokine and cytokine processing to regulate their function. Like the cytokines, MMP expression is downgraded in response to metabolic manipulation (glucosamine) [241]. For example, UVB induction of the collagenases, MMP-1 and MMP-13, was downgraded as a result of short-term glucosamine administration [242]. Though the mechanism(s) by which O-GlcNAc suppresses inflammation-responsive MMPs have not been delineated, MMP transcription factors have been shown to be O-GlcNAc-modified [243]. Investigating the differential O-GlcNAcylation of these transcription factors will be crucial in uncovering how O-GlcNAc signaling regulates MMP expression in inflammation. O-GlcNAc control of MMP expression may have broader implications in mammalian physiology, given the role of MMPs in differentiation, wound-healing and tumorigenesis [244,245,246].

Additional evidence in support of the anti-inflammatory effects of O-GlcNAcylation is exemplified by the effects of enhancing HBP flux on phytohemagglutinin (PHA)-induced inflammation in Jurkat T cells [247]. Glucosamine administration was shown to attenuate IL-2 release in a dose-dependent manner, whereas glucose had a negligible effect on IL-2 levels. This effect was due to the abrogation of NFAT, the cognate IL-2 transcription factor, with metabolic manipulation (glucosamine). The authors provided strong evidence that the subsidence of inflammation is regulated by O-GlcNAc signaling, as the O-GlcNAcase inhibitor, PUGNAc, significantly reduced IL-2 production in the absence of glucosamine. Xing et al. (2011) also demonstrated a role for O-GlcNAc in inflammation resolution [248]. Consistent with other reports, metabolic manipulation (glucosamine) and OGA inhibition (PUGNAc) enhanced O-GlcNAcylation of p65/NFκB in TNFα-induced inflammation; however, in this scenario, NFκB DNA binding and transcriptional activity were impaired in rat aortic smooth muscle cells. Gene expression analysis of the anti-inflammatory mechanism of glucosamine in synovial cells proposed O-GlcNAc-dependent and -independent programs. This conclusion was drawn by comparison of the expression profiles of glucosamine-treated cells exposed to alloxan, a nonspecific OGT inhibitor [249]. The recent development of the highly selective OGT inhibitor series, OSMIs, will aid in clarifying the anti-inflammatory mechanism O-GlcNAc cycling [250].

#### 4.1.2. Integrated Stress Response (ISR)

The integrated stress response (ISR) is a conserved stress-adaptive pathway that enables cells to rapidly adjust the energy-consuming process of translation to better cope with depletion of nutrients, viral injury, accumulation of misfolded proteins, and other sources of intracellular stress [251]. Central to the ISR is the phosphorylation of the translation initiation factor eIF2α by a set of upstream kinases (EIF2AKs: GCN2, HRI, PKR and PERK) that themselves are activated by a variety of extrinsic or intrinsic stress signals [252,253,254,255,256]. Phosphorylation of eIF2α is pivotal in translational regulation, as it attenuates the initiation of general mRNA translation and promotes the translation of mRNAs with upstream ORFs (uORFs), most prominently transcription factors activating transcription factor 4 (ATF4) and CHOP [257,258]. Accumulation of these stress-adaptive transcription factors in turn promotes the expression of other stress responsive genes, including *Atf3, Atf4, Atf5, Gadd34, Gadd45, Chop,* and *Grp78* [259,260,261,262,263,264].

In neuronal cells, increasing UDP-GlcNAc levels via a gain-of-function mutation in GFAT1 (Gly451Glu), or metabolic manipulation (GlcNAc), increased the phosphorylation of PERK and eIF2α as well as promoting the accumulation of transcription factor ATF4 [39]. These changes occurred in the absence of Atf6 cleavage or Xbp1 splicing, indicating that the other branches of the UPR were not implicated in the activation of the eIF2α/ATF4 signaling axis. Ultimately, the induction of the PERK/eIF2α/ATF4 signaling axis was cytoprotective against the accumulation of polyglutamine aggregates by activating their autophagic clearance [39]. Nevertheless, it remains unclear how the elevation of UDP-GlcNAc activates this adaptive ISR response, whether O-GlcNAc is directly implicated, and what O-GlcNAc-modified proteins regulate this signaling pathway. It is noteworthy that eIF2α O-GlcNAcylation counteracts its phosphorylation, with a number of candidate O-GlcNAcylation sites localized within the C-terminus [133]. O-GlcNAcylation of eIF2α would decrease the activation of ISR, which could be beneficial in conditions of excess activation and accumulation of proapoptotic CHOP. Other than eIF2α, the evidence for direct O-GlcNAcylation of other members of the ISR is sparce, with the exception of Chop and Grp78 [265,266].

While O-GlcNAcylation is a regulator of the ISR, existing evidence indicates that the ISR can be upstream of O-GlcNAc cycling. For example, ATF4 transcriptionally activates GFAT1 expression during nutritional stress [267]. In this setting, glucose deprivation activates a GCN2/eIF2α/Atf4 signaling axis that promotes GFAT1 expression, increases HBP flux and ultimately enhances O-GlcNAcylation in cultured human bronchial endothelial cells [267]. In the heart, a similar signaling pathway was identified, whereby stress-induced activation of NADPH-oxidase-4 (Nox4) induces a Nox4/eIF2α/ATF4 signaling axis [268]. ATF4 then increases HBP flux through GFAT1, leading to enhanced O-GlcNAcylation, which in turn promotes pro-adaptive changes in cardiac metabolism that protect against pressure overload stress [268]. Taken together, the evidence points to a nuanced relationship between O-GlcNAcylation and the ISR in which ATF4′s regulation of GFAT1 expression plays a critical role.

In addition to regulation by upstream kinases, the ISR is also impacted by phosphatases acting on the phosphorylation of eIF2α. The main mechanisms involve the recruitment of protein phosphatase 1 (PP1) to eIF2α via scaffold proteins Gadd34 (PPP1R15A) and CReP (PPP1R15B) [269,270]. While existing evidence for the impact of O-GlcNAcylation on subunits of the eIF2α phosphatases is currently scarce, early studies found a physical and functional interaction between subunits of PP1c and OGT or OGA [271,272]. Clearly, more focused studies are needed to determine whether O-GlcNAcylation of catalytic or regulatory subunits of eIF2α phosphatases has an impact on the ISR. In addition to phosphatases, eIF2α phosphorylation is subject to negative regulation by interacting proteins Dnajc3/p58IPK and p67/methionine aminopeptidase 2 (MAP2) [273,274,275]. Regarding the latter, early studies found that p67/MAP2 undergoes O-GlcNAcylation and opposes the phosphorylation of eIF2α by HRI [276,277]. A cluster rich in S/T residues at the N-terminus of p67 is found to undergo O-GlcNAcylation, which is important for its protein stability and regulation of eIF2α phosphorylation [278]. Together, these findings illustrate an alternative mechanism of regulation of the ISR by O-GlcNAcylation where p67/MAP2 binds eIF2α to counter its phosphorylation by EIF2AKs.

#### 4.1.3. O-GlcNAc Cycling Regulates Different Steps in the of Autophagy Pathway

Potentially lethal cellular perturbations can be managed and mitigated by eliminating the insult, preventing the toxic accumulation of damaged macromolecules and dysfunctional organelles via catabolic action or clearance via secretion. Autophagy is a constitutive degradative pathway that contributes to bulk and selective catabolism of intracellular macromolecules and organelles via delivery to the lysosome [279] and also by secretion [280,281]. Autophagic clearance by degradation is particularly advantageous to the cell, as it facilitates metabolite recycling for biosynthesis, in addition to suppressing toxic macromolecule buildup [282,283]. The expanding literature highlights the duality of the autophagy pathway in survival and cell death outcomes as well as the flexibility to adjust to diverse stimuli. Autophagy is noted for its rapid augmentation in response to intracellular and extracellular stress, including nutrient deprivation, proteotoxic stress, inflammation, hypoxia, redox derangement, and microbial stress [284]. The canonical autophagy pathway employs ~40 Atg core proteins in combination with proteins intersecting other pathways to carry out cargo recognition and selection, autophagosome formation with cargo packaging, and ultimately lysosomal delivery for cargo turnover [285]. The considerable number of Atg and non-Atg components are regulated by diverse PTMs, and regulation by O-GlcNAc cycling has been identified at different points in the pathway [285]. It should be noted that many studies report opposing effects on autophagy when global O-GlcNAc levels are up- or downregulated in different cellular contexts. These differences may reflect the heterogeneity in signaling pathway architecture defined by the cell type transcriptional landscape.

The first indications of a role for O-GlcNAc cycling in tuning autophagy stems from altering expression of OGT and OGA in cell culture and whole organism studies [95,286]. OGT-reduced (RNAi) d*rosophila* strains showed enhanced accumulation of autophagic structures by electron microscopy and accumulation of the classic autophagosome marker protein Atg8a, an ortholog of the mammalian LC3, whereas OGT overexpression suppressed formation of autophagic structures and Atg8a abundance [286]. *Ogt-1* and *oga-1* null *C*. *elegans* both demonstrated increased levels of the worm LC3, *lgg-1* [286], and conditional deletion of OGT in cardiac myocytes downregulated LC3-II in the mouse heart and isolated neonatal cardiomyocytes under basal and starved conditions [95]. Molecular focus on the Atg and non-Atg proteins as potential members of the O-GlcNAcome emphasizes the importance of cycling O-GlcNAc on and off individual proteins in regulating different steps in the pathway.

Several of the stress stimuli that activate autophagy converge at the conventional initiation complex composed of ULK1, Atg13, RB1CC1/FIP200 and Atg101 [287]. O-GlcNAc cycling functions at the ULK1 complex, as well as in the post-transcriptional regulation of Atg proteins. The aforementioned *drosophila* study employing OGT-reduced and OGT-overexpressed strains revealed changes in the levels of Atg5 protein, a component of the ATG12 conjugation machinery indispensable for autophagic vesicle formation [286]. OGT overexpression lowered Atg5 abundance, while OGT knockdown increased abundance under fed and fasted conditions. mRNA expression of *Atg5* and *Atg1* (mammalian *Ulk1*) were reduced in the OGT overexpressing strain and enhanced in OGT knockdown strains under basal conditions. The authors assessed the effects of up- and downregulating cellular O-GlcNAc levels on the activity of forkhead box O (FOXO) transcription factors, which positively regulate the expression of several autophagy genes, including *Atg1/Ulk1* and *Atg5* [288,289]. Immunoprecipitation demonstrated O-GlcNAcylation of V5-tagged dFOXO under basal conditions and enhanced O-GlcNAcylation when co-expressed with dOGT in S2 cells. Unsurprisingly, mRNA expression of *Atg1* and *Atg5* was upregulated with dFOXO transfection; however, co-transfection with dOGT suppressed mRNA expression to control levels, suggesting that dFOXO O-GlcNAcylation reduces the transactivation of *Atg1* and *Atg5*. The O-GlcNAcylation sites of the FOXO family of transcription factors may tune target gene selection, as O-GlcNAcylation of mammalian FOXO homologs are associated with transactivation of gluconeogenic and antioxidant genes, as opposed to a general loss of transactivation function [290,291,292].

O-GlcNAcylation of leucyl-tRNA synthetase 1 (LARS1) has been implicated in the regulation of mTORC1, a negative autophagy regulator [293,294]. Glucose promotes the interaction of LARS1 and the RagD GTPase promoting mTORC1 activation [293,294]. Glucose starvation results in the O-GlcNAcylation of LARS1, limiting interaction with RagD and reducing mTORC1 activation by leucine deprivation. Overexpression of an O-GlcNAcylation-deficient LARS1 (S1042A) activated mTORC1 signaling under glucose deprivation, highlighting the role of LARS1 O-GlcNAcylation in the anabolism/catabolism switch [294]. Although autophagy was not directly assessed, it was previously demonstrated that LARS1 binding and phosphorylation by ULK1 was promoted by glucose deprivation and that phosphomimetic mutation at two of the ULK1 phosphorylation sites (Ser2 and Ser720) suppressed LARS1-RagD association and significantly increased autophagosome levels [293]. As such, LARS1 Ser720 phosphorylation may be a sufficient marker for ULK1 activation and autophagy upregulation. OGT knockdown not only reduced LARS1 O-GlcNAcylation under glucose deprivation but also suppressed LARS1 ULK1 interaction and S720 phosphorylation [294]. Loss of ULK1 binding and Ser720 phosphorylation was recapitulated with the O-GlcNAcylation-deficient LARS1, suggesting that pro-autophagic signaling is blunted. This study not only defines a role for LARS1 O-GlcNAcylation in modulating autophagy but also points to another model in which O-GlcNAc functions as a nutrient sensor/metabolism switch.

In contrast to the predicted autophagy upregulation mediated via LARS1 O-GlcNAcylation, O-GlcNAcylation of the pro-autophagic regulator AMPKα in bladder cancer cells was negatively correlated with autophagy activation [295]. Raising global O-GlcNAc levels via OGT overexpression, OGA knockdown and OGA inhibition (TMG) treatment dampened AMPK and ULK1 activation, reducing LC3-II and autophagosomes levels, while the reverse was observed with OGT knockdown and OGA overexpression. Co-transfection of GFP-AMPKα and HA-OGT in bladder cancer cells demonstrated association of the fusion proteins as well as increased O-GlcNAcylation of GFP-AMPKα compared to transfection of GFP-AMPKα alone. O-GlcNAcylation of GFP-AMPKα was also enhanced with OGA inhibition (TMG) treatment relative to the vehicle control [295]. Site information on AMPKα O-GlcNAcylation will be needed to determine whether the changes in AMPKα O-GlcNAcylation represent an increase in O-GlcNAc site stoichiometry or modification of multiple sites on AMPKα.

Recent studies have demonstrated ULK1 O-GlcNAcylation via immunoprecipitation in neonatal mouse cardiomyocytes [95], in a model of starvation-induced liver autophagy [177] and upon lentiviral transduction of a head and neck squamous cell carcinoma (HNSCC) with HPV genes [177]. Elevation of canonical autophagy in these studies was associated with enhanced ULK1 O-GlcNAcylation. As previously mentioned, *Ogt* deletion in mouse cardiac myocytes significantly reduced global O-GlcNAc levels and suppressed autophagic turnover, as indicated by the reduction of LC3-II, persistence of the cargo receptor SQSTM1, and reduced phosphorylation of ATG16L1 under fed and fasted states. In the mouse liver, Ruan et al. [177] also demonstrated suppression of autophagic flux with OGT deletion, suggesting that O-GlcNAcylation is a positive regulator of autophagy in this model. In delineating the pro-autophagic effects associated with O-GlcNAc signaling, the authors showed that ULK1 O-GlcNAcylation during starvation promoted association with the pro-autophagic kinase AMPK and phosphorylation of ULK1 at Ser555, which is known to potentiate ULK1 function. Using an O-GlcNAcylation-inefficient ULK1 (T637A/T754A), the authors demonstrated downregulation of ULK1-AMPK interaction and decreased phosphorylation of ULK1 by AMPK [177].

The co-transduction of HPV16 genes *E6/E7* demonstrated enhanced ULK1 abundance, ULK1 O-GlcNAcylation and accumulation of LC3-II in HNSCC UMSCC17B cells [296]. The higher ULK1 abundance was associated with O-GlcNAcylation, pointing to disruption of ULK1 turnover. The authors assessed the impact of ULK1 O-GlcNAcylation on the recently described CMA turnover of ULK1 [297]. Given that CMA clients are directed to the lysosome via binding the cytosolic chaperone, heat shock cognate 71kDa protein (HSC70) [298], Shi et al. (2022) assessed the ability of wildtype and O-GlcNAc-deficient ULK1 (ULK1-2A) to bind HSC70. The authors demonstrated enhanced HSC70 binding and association of the lysosomal protein LAMP2 by ULK1-2A relative to the wildtype. The increased HSC70 and LAMP2 binding by ULK1-2A suggests that, in this model, O-GlcNAcylation serves as a negative regulator of ULK1 turnover. Inhibition of protein synthesis via cycloheximide treatment revealed the persistence of ULK1-2A, while wildtype ULK1 was turned over in a 6 h period [296]. It is not currently known how protein O-GlcNAcylation regulates selection of other CMA client proteins, but this study opens up a novel area for O-GlcNAc research.

O-GlcNAc controls the protein–protein interactions facilitating membrane fusion, with autophagosome–lysosome fusion being favored by the “de-O-GlcNAcylation” of membrane fusion adaptors SNAP29 and GRASP55 [176,299]. Autophagic turnover of ubiquitinated aggregate-prone proteins utilizes intracellular receptors, including p62/SQSTM1. A suppressor screen for defective receptor (SQST-1) turnover in *C*. *elegans* was rescued by a nonsense mutation in *ogt-1,* which lowered global O-GlcNAc levels. Recapitulation in mammalian cells demonstrated that OGT knockdown enhanced autophagosome-lysosome fusion with the enhanced interaction between autophagosomal SNAP29 and Syntaxin-17 (Stx17) with lysosomal VAMP8 [176]. Both mammalian and *C*. *elegans* SNAP29 are O-GlcNAcylated under basal conditions and deglycosylated under nutrient deprivation and arsenic stress, promoting formation of the SNAP29-Stx17-VAMP complex [176,300]. The Golgi matrix protein GRASP55 is a PDZ domain-containing scaffold protein that makes numerous protein interactions in sorting and compartmentalization of Golgi-resident proteins and cargo, as well as an unconventional secretion pathway-intersecting autophagic components [301,302,303]. Basal conditions maintain GRASP55′s predominantly Golgi localization via mTORC1 phosphorylation [303]. Stress signaling abrogates phosphorylation of GRASP55 by mTORC1, allowing association with multivesicular bodies and autophagosomes, as indicated by colocalization with CHMP2A and LC3B, respectively [303]. Mirroring the localization effects of phosphorylation, GRASP55 is O-GlcNAcylated under basal nutrient conditions and deglycosylated with glucose deprivation, promoting colocalization with the autophagosome [299]. Using an O-GlcNAcylation-deficient GRASP55 (5A), Zhang et al. demonstrated colocalization of WT and 5A GRASP55 at vesicular structures outside the Golgi space with glucose deprivation, whereas glucose refeeding saw WT GRASP55 exhibit significantly reduced vesicular localization while 5A punctate association endured [299].

There are still many possible roles that O-GlcNAc cycling may play in regulating autophagy. The cysteine protein Atg4 is an essential component of the Atg8 conjugation system, which functions in maturation [304] and cargo selection [305,306]. Two independent studies indicate that Atg4 is O-GlcNAc-modified in response to hypoglycemic conditions [294,307]. In addition, the regulation of NFκB function by O-GlcNAc cycling (*previously described*) has yet to be studied in the context of autophagy regulation. Under normoxic conditions, NFκB represses transcription of BCL2/adenovirus E1B 19 kDa protein-interacting protein 3 (BNIP3), a target gene required for hypoxia-induced autophagy [308,309,310]. Perhaps O-GlcNAcylation of the NFκB c-Rel subunit alters promoter selection to activate the transcription of inflammatory cytokines while also de-repressing the BNIP3 promoter under hypoxia.

#### 4.1.4. O-GlcNAcylation and the Pentose Phosphate Pathway (PPP)

The pentose phosphate pathway (PPP) is a critical metabolic circuit that supplies the cell with biosynthetic intermediates and nicotinamide adenine dinucleotide phosphate (NADPH) that serves as a cofactor in biosynthetic reactions and is essential in scavenging ROS. The rate-limiting step in the PPP, the conversion of glucose-6-phosphate to 6-phosphogluconolactone, is catalyzed by the enzyme glucose-6-phoshpate dehydrogenase (G6PD), which is also responsible for the generation of NADPH. In cancer cells, G6PD was found to undergo O-GlcNAcylation, where the modification at Ser84 was essential for maintaining the enzyme’s activity, metabolic flux through the PPP, NADPH production, and increased levels of the ROS scavenger glutathione (GSH) [311]. These features correlated with a growth advantage for tumor cells, and consistently, biopsies of lung human cancers have increased G6PD O-GlcNAcylation [311]. Phosphofructokinase 1 (PFK1) is a rate-limiting enzyme in glycolysis that diverts glucose-6-phosphate away from the PPP and favors its glycolytic conversion to pyruvate. Similarly to G6PD, PFK1 was also found to undergo O-GlcNAcylation in cancer cells [312]. However, in contrast to G6PD, O-GlcNAcylation of PFK1 inhibits enzyme activity. Specifically, enhancing O-GlcNAcylation of PFK1 at Ser259 with OGA inhibition (PUGNAc) or OGT overexpression decreases enzyme activity and glycolysis rates and conversely stimulates flux through the PPP [312]. Furthermore, PFK1 O-GlcNAcylation increases NADPH and GSH levels in cancer cells, and similarly to G6PD, PFK1 O-GlcNAcylation affords a growth advantage to tumor cells [312].

Pyruvate kinase (PKM) catalyzes the conversion of phosphoenolpyruvate to pyruvate, the final rate-limiting step in glycolysis [313]. A splice variant of pyruvate kinase, PKM2, is a ROS-sensitive subtype of pyruvate kinases that, when inhibited, diverts glucose flux from glycolysis to the PPP. Due to its role as a metabolic switch in proliferating cells, the O-GlcNAcylation of PKM2 was investigated. Indeed, PKM2 was O-GlcNAcylated in cancer cells and patient tumors, and two sites (Thr405 and Ser406) were identified as targets for O-GlcNAcylation [314]. O-GlcNAcylation destabilizes PKM2 tetramer formation to reduce enzyme activity and divert glycolytic intermediates to anabolic pathways, including the PPP. Ultimately, PKM2 O-GlcNAcylation in cancer cells promotes tumor proliferation [314]. Following the paradigm of PFK1 and PKM2, the O-GlcNAcylation of phosphoglycerate kinase 1 (PGK1) was investigated in a cancer model. PGK1, which catalyzes the conversion of 1,3-diphosphoglycerate to 3-phosphoglycerate, yielding ATP, was found to be O-GlcNAcylated in cancer cells [315]. Regulatory O-GlcNAcylation of PGK1 occurs at Thr255; however, in contrast to PFK1 and PKM2, O-GlcNAcylation of PGK1 appears to promote its enzyme activity. Nevertheless, similarly to G6PD, PFK1 and PKM2, PGK1 O-GlcNAcylation favors cancer cell proliferation and promotes tumorigenesis [315]. Mechanistically, O-GlcNAcylation promotes PGK1′s activity and favors the translocation of PGK1 to the mitochondria, where it inhibits pyruvate dehydrogenase. Collectively, these events favor the switch from oxidative to glycolytic metabolism that is characteristic of proliferating cancer cells [315]. Together, these studies on O-GlcNAcylation of metabolic enzymes illustrate an emerging theme, where cancer cells employ O-GlcNAcylation to enact metabolic adaptation that enables them to expand during the stressful conditions of cancer proliferation and tumor growth.

## 5. Conclusions and Future Directions

The studies discussed here highlight key roles for O-GlcNAc in regulating metabolism, transcription, translation, signal transduction, and proteostasis, impacting pathways critical to the response to injury and disease pathogenesis (Figure 2). These snapshots support the need for further work in this field that should enhance our understanding of the role of O-GlcNAc in cellular homeostasis, identify novel biomarkers for disease, and highlight pathways that can be manipulated to develop novel therapeutic strategies. To achieve these lofty goals, detection of site-specific O-GlcNAcylation is required, as is the ability to generate proteins that are site-specifically O-GlcNAcylated. Supporting the latter are the recent development of antibodies that enrich O-GlcNAc-modified peptides [316], approaches for introducing site-specific O-GlcNAcylation in vitro and in vivo [195,317], and the development of antibodies that detect site-specific O-GlcNAcylation [161,318].

## Abbreviations

**4E-BP1**: eIF4E binding protein; **Ab** Amyloid-β; **AD**: Alzheimer’s disease; **AGX1/Uap1:** UDP–*N*-acetylglucosamine pyrophosphorylase; **AKT:** Protein kinase B; **ALS:** Amyotrophic Lateral Sclerosis; **Amdhd2:** Amidohydrolase Domain Containing 2; **AMPK:** 5’-AMP-activated protein kinase; **APP:** Amyloid-beta precursor protein; **Atf3-5:** Activating Transcription Factors 3-5; **ATG:** Autophagy-related; **Bag6:** BCL2 Associated Athanogene 6; **BCR:** CD40 and B cell receptor; **BMMs:** Bone marrow derived macrophages; **BNIP3:** BCL2/adenovirus E1B 19 kDa protein-interacting protein 3; **CaMKII:** Calcium/calmodulin-dependent protein kinase II; **CD40:** Tumor necrosis factor receptor superfamily member 5; **CHMP2A:** Charged multivesicular body protein 2a; **CHOP:** CCAAT/Enhancer-Binding Protein Homologous Protein; **CMA:** Chaperone mediated autophagy; **cmOGA-Tg:** Mice with transgenic overexpression of OGA in cardiomyocytes; **Clk:** Cdc2-like kinase; **cmOGT-Tg:** Mice with transgenic overexpression of OGT in cardiomyocytes; **Cox2:** Cyclooxygenase-2; **CPE:** Carboxypeptidase E; **CReP:** Constitutive repressor of eIF2α phosphorylation; **Dnajc3:** DnaJ Heat Shock Protein Family (Hsp40) Member C3; **DON:** Diazo-5-oxo-L-norleucine; **E-C:** Excitation-contraction; **eIF:** Eukaryotic translation initiation factor; **EIF2AK:** Eukaryotic translation initiation factor 2-Alpha Kinase 3; **eIF2α:** Eukaryotic translation initiation factor 2-Alpha; **eIF4E:** Eukaryotic translation initiation factor 4E; **eIF4G1:** Eukaryotic translation initiation factor 4 Gamma, 1; **ER:** Endoplasmic reticulum; **ES:** Embryonic stem; **FASN:** Fatty acid synthase; **FLNA:** Filamin A; **FOXO:** Forkhead Box O family of transcription factors; **fMLP:** N-formyl-methionine-leucine-phenylalanine; **FUS:** Fused in sarcoma; **G6PD:** Glucose-6-phosphate dehydrogenase; **Gadd34:** Growth Arrest And DNA-Damage-Inducible 34; **Gadd45:** Growth Arrest And DNA-Damage-Inducible 45; **GAPDH/*Gapdh*:** Glyceraldehyde-3-phosphate dehydrogenase; **Gata4:** GATA binding protein 4; **GCN2:** General Control Nonderepressible 2; **GFAT (*Gfpt*):** Glutamine:fructose-6 phosphate amidotransferase; **Glut2:** Glucose transporter 2; **Gnpda2**: Glucosamine-6-phosphate deaminase 2; **GNPNAT:** Glucosamine-phosphate N-acetyltransferase; **GPCR:** G-protein coupled receptor; **GRASP55:** Golgi reassembly-stacking protein 2; **Grp78/BiP:** 78 KDa Glucose Regulated Protein; **GSH:** Glutathione; **GSK3β**—Glycogen Synthase Kinase β; **Gsta2:** Glutathione S-Transferase Alpha 2; **GTPase:** Rac1 guanosine triphosphatase; **HAT:** Histone acetyltransferase; **HBP:** Hexosamine biosynthetic pathway; **HPV:** Human papillomavirus; **HCF1:** Host cell factor 1; **Hif1α:** Hypoxia inducible factor 1 alpha; **HNSCC:** Head and neck squamous cell carcinoma; **HRI:** Heme-Regulated Inhibitor; **Hsc70:** Heat shock cognate 71 KDa Protein; **HSP:** Heat shock protein; **I/R:** Ischemia-reperfusion; **IKK:** I-kappa B kinase complex; **IL-6/Il6:** Interleukin 6; **iPSC:** Induced pluripotent stem cell; **ISO:** Isoproterenol; **ISR:** Integrated stress response; **LARS1:** Leucyl-tRNA synthetase 1; **IκBα:** NF-kappa-B inhibitor alpha; **JAK-STAT:** Janus kinase-signal transducer and activator of transcription; **LC3:** Microtubule-associated proteins 1A/1B light chain 3; **LPS:** Lipopolysaccharide; **MAP2:** Methionine aminopeptidase 2; **MAPK:** Mitogen activated protein kinase; **MEFs:** Mouse Embryo Fibroblasts; **MI:** Myocardial infarction; **miRs:** MicroRNAs; **MKK:** Mitogen-activated kinase kinases; **MMPs:** Matrix metalloproteinases; **mPTP:** Mitochondrial permeability transition pore; **mTORC1:** Mechanistic target of rapamycin complex 1; **Myh6:** Myosin heavy chain 6; ***Myh7:*** Myosin heavy chain 7; **NADPH:** Nicotinamide adenine dinucleotide phosphate; **NButGT:** 1,2-dideoxy-2′-propyl-α-d-glucopyranoso-[2,1-d]-Δ2′-thiazoline; **NF-κB:** Nuclear factor kappa B subunit 1; **NFAT:** Nuclear factor of activated T cells; **NFT:** Neurofibrillary tangle; **Nkx2-5:** NK2 Transcription Factor Related, Locus 5; **NLRP3:** NOD-like receptor (NLR) family pyrin domain-containing 3; **NPGPx/GPx7:** nonselenocysteine-containing phospholipid hydroperoxide glutathione peroxidase/Glutathione peroxidase 7; **Nox4:** NADPH oxidase 4; ***Nppa:*** Natriuretic peptide alpha; **O-GlcNAc:** O-linked b N-acetylglucosamine; **OGA:** O-GlcNAcase; **I-cmOGT KO:** Inducible adult-onset OGT cardiomyocyte deletion; **OGT:** O-GlcNAc transferase; **OSMI-1:** (R)-N-(furan-2- ylmethyl)-2-(2-methoxyphenyl)-2-(2-oxo-1,2-dihydroquinoline-6-sulfonamido)-N-(thiophen-2-ylmethyl)acetamide; **Orai1:** Pronounced as ‘Horae’ mythological keepers of the gates of heaven; **OSMI;** OGT small molecule inhibitor; **p58IPK:** 58 KDa Protein Kinase Inhibitor; **PABP1:** Poly(A)-Binding Protein 1; **PDI:** Protein disulfide isomerase; **PDZ:** Combination of the first letters of the first three proteins discovered to share the domain—post synaptic density protein (PSD95), Drosophila disc large tumor suppressor (Dlg1), and zonula occludens-1 protein (zo-1); **PERK:** PKR-like ER kinase; **PFK1:** Phosphofructokinase 1; **PGC1α:** Peroxisome Proliferative Activated Receptor, Gamma, Coactivator 1, Alpha; **PGK1:** Phosphoglycerate kinase 1; **PGM3:** Phosphoglucomutase 3; **PHA:** Phytohemagglutinin; **PI3K:** Phosphatidylinositol-3-kinase; **PKA:** cAMP-dependent protein kinase; **PKM2:** Pyruvate kinase splice isoform 2; **PKR:** Protein Kinase R; **PMN:** Polymorphonuclear leukocyte; **PO:** Pressure overload; **PP1; Protein** Phosphatase 1; **PPP:** Pentose phosphate pathway; **PPP1R15A:** Protein Phosphatase 1, Regulatory Subunit 15A; **PPP1R15B:** Protein Phosphatase 1, Regulatory Subunit 15B; **PRMT1:** Protein Arginine Methyltransferase 1; **PRMT4/Carm1:** Protein Arginine Methyltransferase 4; **PRMT5:** Protein arginine methyltransferase 5; **PTM** Post-translational modification; **PUGNAc:** O-(2-Acetamido-2-deoxy-D-glucopyranosylidenamino) N-phenylcarbamate; **Rac1:** Ras-related C3 botulinum toxin substrate 1; **RagD:** Ras-related GTP-binding protein D; **RASMC:** Rat aortic smooth muscle cell; **RB1CC1/FIP200:** RB1-inducible coiled-coil protein 1/FAK family kinase-interacting protein of 200 kDa; **RelA/p65:** RelA protooncogene, NFkB subunit; **RIPK:** Receptor-interacting serine/threonine kinase; **ROS:** Reactive oxygen species; **RyR:** Ryanodine receptor; **sAPPα:** Soluble fragment of APPa; **sAPPβ:** Soluble fragment APPb; **SR:** Sarcoplasmic reticulum; **SERCA2A:** Sarcoplasmic/endoplasmic reticulum calcium ATPase 2; **SNAP29:** Synaptosomal-associated protein 29; **SOD1:** Superoxide dismutase 1; **STIM1:** Stromal Interaction Molecule 1; **Stx17:** Syntaxin-17; **TAB1:** TAK1-binding protein 1; **TAK1:** Transforming growth factor β-activated kinase 1; **TDP43:** Transactive response DNA binding protein 43; **TGFβ:** Transforming growth factor β; **TH:** Traumatic hemorrhage; **TMG:** Thiamet G; **TNFα/*Tnfa*:** Tumor necrosis factor-alpha; **Tnks1bp1:** Tankyrase 1 binding protein 1; **TPR:** Tetratricopeptide repeat; **UDP-GlcNAc:** Uridine-5′-diphosphate-N-acetylglucosamine; **uORFs:** upstream ORF; **ULK1:** Unc-51 like autophagy activating kinase 1; **UPR:** Unfolded protein response; **VAMP8:** Vesicle-associated membrane protein 8; **VDAC:** Voltage dependent anion channel; **Xbp1s:** X-Box-binding protein 1-spliced.

## References

[1] Hart G.W. (2019). Nutrient Regulation of Signaling and Transcription. J. Biol. Chem..

[2] Ma J., Li Y., Hou C., Wu C. (2021). O-GlcNAcAtlas: A Database of Experimentally Identified O-GlcNAc Sites and Proteins. Glycobiology.

[3] Wulff-Fuentes E., Berendt R.R., Massman L., Danner L., Malard F., Vora J., Kahsay R., Stichelen S.O.-V. (2020). The Human O-GlcNAcome Database and Meta-Analysis. Sci. Data.

[4] Gewinner C., Hart G., Zachara N., Cole R., Beisenherz-Huss C., Groner B. (2004). The Coactivator of Transcription CREB-Binding Protein Interacts Preferentially with the Glycosylated Form of Stat5. J. Biol. Chem..

[5] Lamarre-Vincent N., Hsieh-Wilson L.C. (2003). Dynamic Glycosylation of the Transcription Factor CREB: A Potential Role in Gene Regulation. J. Am. Chem. Soc..

[6] Deplus R., Delatte B., Schwinn M.K., Defrance M., Méndez J., Murphy N., Dawson M.A., Volkmar M., Putmans P., Calonne E. (2013). TET2 and TET3 Regulate GlcNAcylation and H3K4 Methylation through OGT and SET1/COMPASS. EMBO J..

[7] Slawson C., Zachara N.E., Vosseller K., Cheung W.D., Lane M.D., Hart G.W. (2005). Perturbations in O-Linked β-N-Acetylglucosamine Protein Modification Cause Severe Defects in Mitotic Progression and Cytokinesis. J. Biol. Chem..

[8] Zhu Y., Liu T.-W., Cecioni S., Eskandari R., Zandberg W.F., Vocadlo D.J. (2015). O-GlcNAc Occurs Cotranslationally to Stabilize Nascent Polypeptide Chains. Nat. Chem. Biol..

[9] Zhu Y., Liu T., Madden Z., Yuzwa S.A., Murray K., Cecioni S., Zachara N., Vocadlo D.J. (2015). Post-Translational O-GlcNAcylation Is Essential for Nuclear Pore Integrity and Maintenance of the Pore Selectivity Filter. J. Mol. Cell Biol..

[10] Sümegi M., Hunyadi-Gulyás E., Medzihradszky K.F., Udvardy A. (2003). 26S Proteasome Subunits Are O-Linked N-Acetylglucosamine-Modified in Drosophila Melanogaster. Biochem. Biophys. Res. Commun..

[11] Yoo T.Y., Mitchison T.J. (2021). O-GlcNAc Modification of Nuclear Pore Complexes Accelerates Bidirectional Transport. J. Cell Biol..

[12] Yang X., Su K., Roos M.D., Chang Q., Paterson A.J., Kudlow J.E. (2001). O-Linkage of N-Acetylglucosamine to Sp1 Activation Domain Inhibits Its Transcriptional Capability. Proc. Natl. Acad. Sci. USA.

[13] Toleman C.A., Schumacher M.A., Yu S.-H., Zeng W., Cox N.J., Smith T.J., Soderblom E.J., Wands A.M., Kohler J.J., Boyce M. (2018). Structural Basis of O-GlcNAc Recognition by Mammalian 14-3-3 Proteins. Proc. Natl. Acad. Sci. USA.

[14] Dias W.B., Cheung W.D., Wang Z., Hart G.W. (2009). Regulation of Calcium/Calmodulin-Dependent Kinase IV by O-GlcNAc Modification. J. Biol. Chem..

[15] Pathak S., Borodkin V.S., Albarbarawi O., Campbell D.G., Ibrahim A., van Aalten D.M. (2012). O-GlcNAcylation of TAB1 Modulates TAK1-Mediated Cytokine Release. EMBO J..

[16] Tarrant M.K., Rho H.-S., Xie Z., Jiang Y.L., Gross C., Culhane J.C., Yan G., Qian J., Ichikawa Y., Matsuoka T. (2012). Regulation of CK2 by Phosphorylation and O-GlcNAcylation Revealed by Semisynthesis. Nat. Chem. Biol..

[17] Zachara N.E., O’Donnell N., Cheung W.D., Mercer J.J., Marth J.D., Hart G.W. (2004). Dynamic O-GlcNAc Modification of Nucleocytoplasmic Proteins in Response to Stress. J. Biol. Chem..

[18] Kazemi Z., Chang H., Haserodt S., McKen C., Zachara N.E. (2010). O-Linked-N-Acetylglucosamine (O-GlcNAc) Regulates Stress-Induced Heat Shock Protein Expression in a GSK-3 -Dependent Manner. J. Biol. Chem..

[19] Jones S.P., Zachara N.E., Ngoh G.A., Hill B.G., Teshima Y., Bhatnagar A., Hart G.W., Marbán E. (2008). Cardioprotection by N-Acetylglucosamine Linkage to Cellular Proteins. Circulation.

[20] Karababa A., Görg B., Schliess F., Häussinger D. (2014). O-GlcNAcylation as a Novel Ammonia-Induced Posttranslational Protein Modification in Cultured Rat Astrocytes. Metab. Brain Dis..

[21] Al-Mukh H., Baudoin L., Bouaboud A., Sanchez-Salgado J.-L., Maraqa N., Khair M., Pagesy P., Bismuth G., Niedergang F., Issad T. (2020). Lipopolysaccharide Induces GFAT2 Expression to Promote O-Linked β-N-Acetylglucosaminylation and Attenuate Inflammation in Macrophages. J. Immunol..

[22] Ngoh G.A., Facundo H.T., Hamid T., Dillmann W., Zachara N.E., Jones S.P. (2008). Unique Hexosaminidase Reduces Metabolic Survival Signal and Sensitizes Cardiac Myocytes to Hypoxia/Reoxygenation Injury. Circ. Res..

[23] Zhu W.Z., Ledee D., Olson A.K. (2021). Temporal Regulation of Protein O-GlcNAc Levels during Pressure-overload Cardiac Hypertrophy. Physiol. Rep..

[24] Zou L., Yang S., Hu S., Chaudry I.H., Marchase R.B., Chatham J.C. (2007). The Protective Effects of PUGNAc on Cardiac Function after Trauma-Hemorrhage Are Mediated via Increased Protein O-GlcNAc Levels. Shock.

[25] Lee A., Miller D., Henry R., Paruchuri V.D.P., O’Meally R.N., Boronina T., Cole R.N., Zachara N.E. (2016). Combined Antibody/Lectin Enrichment Identifies Extensive Changes in the O-GlcNAc Sub-Proteome upon Oxidative Stress. J. Proteome Res..

[26] Haltiwanger R.S., Holt G.D., Hart G.W. (1990). Enzymatic Addition of O-GlcNAc to Nuclear and Cytoplasmic Proteins. Identification of a Uridine Diphospho-N-Acetylglucosamine:Peptide Beta-N-Acetylglucosaminyltransferase. J. Biol. Chem..

[27] Gao Y., Wells L., Comer F.I., Parker G.J., Hart G.W. (2001). Dynamic O-Glycosylation of Nuclear and Cytosolic Proteins: Cloning and Characterization of a Neutral, Cytosolic Beta-N-Acetylglucosaminidase from Human Brain. J. Biol. Chem..

[28] Olson A.K., Bouchard B., Zhu W.Z., Chatham J.C., Rosiers C.D. (2020). First Characterization of Glucose Flux through the Hexosamine Biosynthesis Pathway (HBP) in Ex Vivo Mouse Heart. J. Biol. Chem..

[29] Kornfeld S., Kornfeld R., Neufeld E.F., O’Brien P.J. (1964). The Feedback Control of Sugar Nucleotide Biosynthesis in Liver. Proc. Natl. Acad. Sci. USA.

[30] Kornfeld R. (1967). Studies on L-Glutamine D-Fructose 6-Phosphate Amidotransferase. I. Feedback Inhibition by Uridine Diphosphate-N-Acetylglucosamine. J. Biological. Chem..

[31] Eguchi S., Oshiro N., Miyamoto T., Yoshino K.-I., Okamoto S., Ono T., Kikkawa U., Yonezawa K. (2009). AMP-Activated Protein Kinase Phosphorylates Glutamine: Fructose-6-Phosphate Amidotransferase 1 at Ser243 to Modulate Its Enzymatic Activity. Genes Cells.

[32] Li Y., Roux C., Lazereg S., LeCaer J.-P., Laprévote O., Badet B., Badet-Denisot M.-A. (2007). Identification of a Novel Serine Phosphorylation Site in Human Glutamine:Fructose-6-Phosphate Amidotransferase Isoform 1. Biochemistry.

[33] Hu Y., Riesland L., Paterson A.J., Kudlow J.E. (2004). Phosphorylation of Mouse Glutamine-Fructose-6-Phosphate Amidotransferase 2 (GFAT2) by CAMP-Dependent Protein Kinase Increases the Enzyme Activity. J. Biol. Chem..

[34] Gutierrez-Aguilar R., Grayson B.E., Kim D.-H., Yalamanchili S., Calcagno M.L., Woods S.C., Seeley R.J. (2021). CNS GNPDA2 Does Not Control Appetite, but Regulates Glucose Homeostasis. Front. Nutr..

[35] Kroef V., Ruegenberg S., Horn M., Allmeroth K., Ebert L., Bozkus S., Miethe S., Elling U., Schermer B., Baumann U. (2022). GFPT2/GFAT2 and AMDHD2 Act in Tandem to Control the Hexosamine Pathway. eLife.

[36] Freeze H.H., Boyce M., Zachara N.E., Hart G.W., Schnaar R.L., Varki A., Cummings R.D., Esko J.D., Stanley P., Hart G.W., Aebi M., Mohnen D., Kinoshita T., Packer N.H., Prestegard J.H. (2022). Glycosylation Precursors.

[37] Ryczko M.C., Pawling J., Chen R., Rahman A.M.A., Yau K., Copeland J.K., Zhang C., Surendra A., Guttman D.S., Figeys D. (2016). Metabolic Reprogramming by Hexosamine Biosynthetic and Golgi N-Glycan Branching Pathways. Sci. Rep..

[38] Wang Z.V., Deng Y., Gao N., Pedrozo Z., Li D.L., Morales C.R., Criollo A., Luo X., Tan W., Jiang N. (2014). Spliced X-Box Binding Protein 1 Couples the Unfolded Protein Response to Hexosamine Biosynthetic Pathway. Cell.

[39] Horn M., Denzel S.I., Srinivasan B., Allmeroth K., Schiffer I., Karthikaisamy V., Miethe S., Breuer P., Antebi A., Denzel M.S. (2020). Hexosamine Pathway Activation Improves Protein Homeostasis through the Integrated Stress Response. Iscience.

[40] Ruegenberg S., Horn M., Pichlo C., Allmeroth K., Baumann U., Denzel M.S. (2020). Loss of GFAT-1 Feedback Regulation Activates the Hexosamine Pathway That Modulates Protein Homeostasis. Nat. Commun..

[41] Taparra K., Wang H., Malek R., Lafargue A., Barbhuiya M.A., Wang X., Simons B.W., Ballew M., Nugent K., Groves J. (2018). O-GlcNAcylation Is Required for Mutant KRAS-Induced Lung Tumorigenesis. J. Clin. Investig..

[42] Itkonen H.M., Engedal N., Babaie E., Luhr M., Guldvik I.J., Minner S., Hohloch J., Tsourlakis M.C., Schlomm T., Mills I.G. (2014). UAP1 Is Overexpressed in Prostate Cancer and Is Protective against Inhibitors Of. Nat. Publ. Group.

[43] Capotosti F., Guernier S., Lammers F., Waridel P., Cai Y., Jin J., Conaway J.W., Conaway R.C., Herr W. (2011). O-GlcNAc Transferase Catalyzes Site-Specific Proteolysis of HCF-1. Cell.

[44] Daou S., Mashtalir N., Hammond-Martel I., Pak H., Yu H., Sui G., Vogel J.L., Kristie T.M., Affar E.B. (2011). Crosstalk between O-GlcNAcylation and Proteolytic Cleavage Regulates the Host Cell Factor-1 Maturation Pathway. Proc. Natl. Acad. Sci. USA.

[45] Levine Z.G., Potter S.C., Joiner C.M., Fei G.Q., Nabet B., Sonnett M., Zachara N.E., Gray N.S., Paulo J.A., Walker S. (2021). Mammalian Cell Proliferation Requires Noncatalytic Functions of O-GlcNAc Transferase. Proc. Natl. Acad. Sci. USA.

[46] Watson L.J., Facundo H.T., Ngoh G.A., Ameen M., Brainard R.E., Lemma K.M., Long B.W., Prabhu S.D., Xuan Y.-T., Jones S.P. (2010). O-Linked β-N-Acetylglucosamine Transferase Is Indispensable in the Failing Heart. Proc. Natl. Acad. Sci. USA.

[47] Watson L.J., Long B.W., DeMartino A.M., Brittian K.R., Readnower R.D., Brainard R.E., Cummins T.D., Annamalai L., Hill B.G., Jones S.P. (2014). Cardiomyocyte Ogt Is Essential for Postnatal Viability. AJP Heart Circ. Physiol..

[48] Zhang B., Li M.-D., Yin R., Liu Y., Yang Y., Mitchell-Richards K.A., Nam J.H., Li R., Wang L., Iwakiri Y. (2019). O-GlcNAc Transferase Suppresses Necroptosis and Liver Fibrosis. JCI Insight.

[49] Levine Z.G., Fan C., Melicher M.S., Orman M., Benjamin T., Walker S. (2018). O-GlcNAc Transferase Recognizes Protein Substrates Using an Asparagine Ladder in the Tetratricopeptide Repeat (TPR) Superhelix. J. Am. Chem. Soc..

[50] Joiner C.M., Levine Z.G., Aonbangkhen C., Woo C.M., Walker S. (2019). Aspartate Residues Far from the Active Site Drive O-GlcNAc Transferase Substrate Selection. J. Am. Chem. Soc..

[51] Joiner C.M., Hammel F.A., Janetzko J., Walker S. (2021). Protein Substrates Engage the Lumen of O-GlcNAc Transferase’s Tetratricopeptide Repeat Domain in Different Ways. Biochemistry.

[52] Martinez M., Renuse S., Kreimer S., O’Meally R., Natov P., Madugundu A.K., Nirujogi R.S., Tahir R., Cole R., Pandey A. (2021). Quantitative Proteomics Reveals That the OGT Interactome Is Remodeled in Response to Oxidative Stress. Mol. Cell Proteom..

[53] Cheung W.D., Sakabe K., Housley M.P., Dias W.B., Hart G.W. (2008). O-Linked-N-Acetylglucosaminyltransferase Substrate Specificity Is Regulated by Myosin Phosphatase Targeting and Other Interacting Proteins. J. Biol. Chem..

[54] Cheung W.D., Hart G.W. (2008). AMP-Activated Protein Kinase and P38 MAPK Activate O-GlcNAcylation of Neuronal Proteins during Glucose Deprivation. J. Biol. Chem..

[55] Groves J.A., Maduka A.O., O’Meally R.N., Cole R.N., Zachara N.E. (2017). Fatty Acid Synthase Inhibits the O-GlcNAcase during Oxidative Stress. J. Biol. Chem..

[56] Jensen R.V., Zachara N.E., Nielsen P.H., Kimose H.H., Kristiansen S.B., Bøtker H.E. (2013). Impact of O-GlcNAc on Cardioprotection by Remote Ischaemic Preconditioning in Non-Diabetic and Diabetic Patients. Cardiovasc. Res..

[57] Zhong J., Martinez M., Sengupta S., Lee A., Wu X., Chaerkady R., Chatterjee A., O’Meally R.N., Cole R.N., Pandey A. (2015). Quantitative Phosphoproteomics Reveals Crosstalk Between Phosphorylation and O-GlcNAc in the DNA Damage Response Pathway. Proteomics.

[58] Jensen R.V., Johnsen J., Kristiansen S.B., Zachara N.E., Bøtker H.E. (2013). Ischemic Preconditioning Increases Myocardial O-GlcNAc Glycosylation. Scand. Cardiovasc. J..

[59] Ryu I.-H., Do S.-I. (2011). Denitrosylation of S-Nitrosylated OGT Is Triggered in LPS-Stimulated Innate Immune Response. Biochem. Biophys. Res. Commun..

[60] Seo H.G., Kim H.B., Yoon J.Y., Kweon T.H., Park Y.S., Kang J., Jung J., Son S., Yi E.C., Lee T.H. (2020). Mutual Regulation between OGT and XIAP to Control Colon Cancer Cell Growth and Invasion. Cell Death Dis..

[61] Kaasik K., Kivimäe S., Allen J.J., Chalkley R.J., Huang Y., Baer K., Kissel H., Burlingame A.L., Shokat K.M., Ptáček L.J. (2013). Glucose Sensor O-GlcNAcylation Coordinates with Phosphorylation to Regulate Circadian Clock. Cell Metab..

[62] Rao F.V., Dorfmueller H.C., Villa F., Allwood M., Eggleston I.M., van Aalten D.M.F. (2006). Structural Insights into the Mechanism and Inhibition of Eukaryotic O-GlcNAc Hydrolysis. EMBO J..

[63] Schimpl M., Borodkin V.S., Gray L.J., van Aalten D.M.F. (2012). Synergy of Peptide and Sugar in O-GlcNAcase Substrate Recognition. Chem. Biol..

[64] Li B., Li H., Hu C.-W., Jiang J. (2017). Structural Insights into the Substrate Binding Adaptability and Specificity of Human O-GlcNAcase. Nat. Commun..

[65] Schultz J., Pils B. (2002). Prediction of Structure and Functional Residues for O-GlcNAcase, a Divergent Homologue of Acetyltransferases. FEBS Lett..

[66] Elsen N.L., Patel S.B., Ford R.E., Hall D.L., Hess F., Kandula H., Kornienko M., Reid J., Selnick H., Shipman J.M. (2017). Insights into Activity and Inhibition from the Crystal Structure of Human O-GlcNAcase. Nat. Chem. Biol..

[67] Keembiyehetty C.N., Krzeslak A., Love D.C., Hanover J.A. (2011). A Lipid-Droplet-Targeted O-GlcNAcase Isoform Is a Key Regulator of the Proteasome. J. Cell Sci..

[68] Pagesy P., Bouaboud A., Feng Z., Hulin P., Issad T. (2022). Short O-GlcNAcase Is Targeted to the Mitochondria and Regulates Mitochondrial Reactive Oxygen Species Level. Cells.

[69] Dontaine J., Bouali A., Daussin F., Bultot L., Vertommen D., Martin M., Rathagirishnan R., Cuillerier A., Horman S., Beauloye C. (2022). The Intra-Mitochondrial O-GlcNAcylation System Rapidly Modulates OXPHOS Function and ROS Release in the Heart. Commun. Biol..

[70] Wells L., Gao Y., Mahoney J.A., Vosseller K., Chen C., Rosen A., Hart G.W. (2002). Dynamic O-Glycosylation of Nuclear and Cytosolic Proteins: Further Characterization of the Nucleocytoplasmic Beta-N-Acetylglucosaminidase, O-GlcNAcase. J. Biol. Chem..

[71] Butkinaree C., Cheung W.D., Park S., Park K., Barber M., Hart G.W. (2008). Characterization of Beta-N-Acetylglucosaminidase Cleavage by Caspase-3 during Apoptosis. J. Biol. Chem..

[72] Wagner S.A., Beli P., Weinert B.T., Nielsen M.L., Cox J., Mann M., Choudhary C. (2011). A Proteome-Wide, Quantitative Survey of in Vivo Ubiquitylation Sites Reveals Widespread Regulatory Roles. Mol. Cell. Proteom..

[73] Biarc J., Chalkley R.J., Burlingame A.L., Bradshaw R.A. (2012). The Induction of Serine/Threonine Protein Phosphorylations by a PDGFR/TrkA Chimera in Stably Transfected PC12 Cells. Mol. Cell. Proteom..

[74] Udeshi N.D., Svinkina T., Mertins P., Kuhn E., Mani D.R., Qiao J.W., Carr S.A. (2013). Refined Preparation and Use of Anti-Diglycine Remnant (K-ε-GG) Antibody Enables Routine Quantification of 10,000s of Ubiquitination Sites in Single Proteomics Experiments*. Mol. Cell. Proteom..

[75] Muthusamy S., DeMartino A.M., Watson L.J., Brittian K.R., Zafir A., Dassanayaka S., Hong K.U., Jones S.P. (2014). MicroRNA-539 Is up-Regulated in Failing Heart, and Suppresses O-GlcNAcase Expression. J. Biol. Chem..

[76] Yan W., Cao M., Ruan X., Jiang L., Lee S., Lemanek A., Ghassemian M., Pizzo D.P., Wan Y., Qiao Y. (2022). Cancer-Cell-Secreted MiR-122 Suppresses O-GlcNAcylation to Promote Skeletal Muscle Proteolysis. Nat. Cell Biol..

[77] Park S.-K., Zhou X., Pendleton K.E., Hunter O.V., Kohler J.J., O’Donnell K.A., Conrad N.K. (2017). A Conserved Splicing Silencer Dynamically Regulates O-GlcNAc Transferase Intron Retention and O-GlcNAc Homeostasis. Cell Rep..

[78] Tan Z.-W., Fei G., Paulo J.A., Bellaousov S., Martin S.E.S., Duveau D.Y., Thomas C.J., Gygi S.P., Boutz P.L., Walker S. (2020). O-GlcNAc Regulates Gene Expression by Controlling Detained Intron Splicing. Nucleic Acids Res..

[79] Ninomiya K., Kataoka N., Hagiwara M. (2011). Stress-Responsive Maturation of Clk1/4 Pre-MRNAs Promotes Phosphorylation of SR Splicing Factor. J. Cell Biol..

[80] Li Z., Xu J., Song Y., Xin C., Liu L., Hou N., Teng Y., Cheng X., Wang T., Yu Z. (2021). PRMT5 Prevents Dilated Cardiomyopathy via Suppression of Protein O-GlcNAcylation. Circ. Res..

[81] Schimpl M., Schüttelkopf A.W., Borodkin V.S., van Aalten D.M.F. (2010). Human OGA Binds Substrates in a Conserved Peptide Recognition Groove. Biochem. J..

[82] Stephen H.M., Praissman J.L., Wells L. (2021). Generation of an Interactome for the Tetratricopeptide Repeat Domain of O-GlcNAc Transferase Indicates a Role for the Enzyme in Intellectual Disability. J. Proteome Res..

[83] O’Donnell N., Zachara N.E., Hart G.W., Marth J.D. (2004). Ogt-Dependent X-Chromosome-Linked Protein Glycosylation Is a Requisite Modification in Somatic Cell Function and Embryo Viability. Mol. Cell. Biol..

[84] Shafi R., Iyer S.P., Ellies L.G., O’Donnell N., Marek K.W., Chui D., Hart G.W., Marth J.D. (2000). The O-GlcNAc Transferase Gene Resides on the X Chromosome and Is Essential for Embryonic Stem Cell Viability and Mouse Ontogeny. Proc. Natl. Acad. Sci. USA.

[85] Mu Y., Yu H., Wu T., Zhang J., Evans S.M., Chen J. (2020). O-Linked β-N-Acetylglucosamine Transferase Plays an Essential Role in Heart Development through Regulating Angiopoietin-1. PLoS Genet..

[86] Kim H.-S., Park S.Y., Choi Y.R., Kang J.G., Joo H.J., Moon W.K., Cho J.W. (2009). Excessive O-GlcNAcylation of Proteins Suppresses Spontaneous Cardiogenesis in ES Cells. FEBS Lett..

[87] Kim H.S., Woo J.S., Joo H.J., Moon W.K. (2012). Cardiac Transcription Factor Nkx2.5 Is Downregulated under Excessive O-GlcNAcylation Condition. PLoS ONE.

[88] Jang H., Kim T.W., Yoon S., Choi S.-Y., Kang T.-W., Kim S.-Y., Kwon Y.-W., Cho E.-J., Youn H.-D. (2012). O-GlcNAc Regulates Pluripotency and Reprogramming by Directly Acting on Core Components of the Pluripotency Network. Cell Stem Cell.

[89] Speakman C.M., Domke T.C.E., Wongpaiboonwattana W., Sanders K., Mudaliar M., van Aalten D.M.F., Barton G.J., Stavridis M.P. (2014). Elevated O-GlcNAc Levels Activate Epigenetically Repressed Genes and Delay Mouse ESC Differentiation Without Affecting Naïve to Primed Cell Transition. Stem Cells.

[90] Hao Y., Fan X., Shi Y., Zhang C., Sun D.-E., Qin K., Qin W., Zhou W., Chen X. (2019). Next-Generation Unnatural Monosaccharides Reveal That ESRRB O-GlcNAcylation Regulates Pluripotency of Mouse Embryonic Stem Cells. Nat. Commun..

[91] Zafir A., Bradley J.A., Long B.W., Muthusamy S., Li Q., Hill B.G., Wysoczynski M., Prabhu S.D., Bhatnagar A., Bolli R. (2015). O-GlcNAcylation Negatively Regulates Cardiomyogenic Fate in Adult Mouse Cardiac Mesenchymal Stromal Cells. PLoS ONE.

[92] Umapathi P., Mesubi O.O., Banerjee P.S., Abrol N., Wang Q., Luczak E.D., Wu Y., Granger J.M., Wei A.-C., Gaido O.E.R. (2021). Excessive O-GlcNAcylation Causes Heart Failure and Sudden Death. Circulation.

[93] Ma J., Banerjee P., Whelan S.A., Liu T., Wei A.-C., Ramirez-Correa G., McComb M.E., Costello C.E., O’Rourke B., Murphy A. (2016). Comparative Proteomics Reveals Dysregulated Mitochondrial O-GlcNAcylation in Diabetic Hearts. J. Proteome Res..

[94] Ma J., Liu T., Wei A.-C., Banerjee P., O’Rourke B., Hart G.W. (2015). O-GlcNAcomic Profiling Identifies Widespread O-Linked β-N-Acetylglucosamine Modification (O-GlcNAcylation) in Oxidative Phosphorylation System Regulating Cardiac Mitochondrial Function* ^♦^. J. Biol. Chem..

[95] Yu H., Wen L., Mu Y. (2020). O-GlcNAcylation Is Essential for Autophagy in Cardiomyocytes. Oxidative Med. Cell. Longev..

[96] Ednie A.R., Bennett E.S. (2020). Intracellular O-Linked Glycosylation Directly Regulates Cardiomyocyte L-Type Ca^2+^ Channel Activity and Excitation–Contraction Coupling. Basic Res. Cardiol..

[97] Erickson J.R., Pereira L., Wang L., Han G., Ferguson A., Dao K., Copeland R.J., Despa F., Hart G.W., Ripplinger C.M. (2013). Diabetic Hyperglycaemia Activates CaMKII and Arrhythmias by O-Linked Glycosylation. Nature.

[98] Hegyi B., Fasoli A., Ko C.Y., Van B.W., Alim C.C., Shen E.Y., Ciccozzi M.M., Tapa S., Ripplinger C.M., Erickson J.R. (2021). CaMKII Serine 280 O-GlcNAcylation Links Diabetic Hyperglycemia to Proarrhythmia. Circ. Res..

[99] Zhu-Mauldin X., Marsh S.A., Zou L., Marchase R.B., Chatham J.C. (2012). Modification of STIM1 by O-Linked N-Acetylglucosamine (O-GlcNAc) Attenuates Store-Operated Calcium Entry in Neonatal Cardiomyocytes*. J. Biol. Chem..

[100] Nomura A., Yokoe S., Tomoda K., Nakagawa T., Martin-Romero F.J., Asahi M. (2020). Fluctuation in O-GlcNAcylation Inactivates STIM1 to Reduce Store-Operated Calcium Ion Entry via down-Regulation of Ser621 Phosphorylation. J. Biological. Chem..

[101] Keembiyehetty C., Love D.C., Harwood K.R., Gavrilova O., Comly M.E., Hanover J.A. (2015). Conditional Knock-out Reveals a Requirement for O-Linked N-Acetylglucosaminase (O-GlcNAcase) in Metabolic Homeostasis. J. Biol. Chem..

[102] Yang Y.R., Song M., Lee H., Jeon Y., Choi E.-J., Jang H.-J., Moon H.Y., Byun H.-Y., Kim E.-K., Kim D.H. (2012). O-GlcNAcase Is Essential for Embryonic Development and Maintenance of Genomic Stability. Aging Cell.

[103] Muha V., Authier F., Szoke-Kovacs Z., Johnson S., Gallagher J., McNeilly A., McCrimmon R.J., Teboul L., van Aalten D.M.F. (2021). Loss of O-GlcNAcase Catalytic Activity Leads to Defects in Mouse Embryogenesis. J. Biological. Chem..

[104] Dassanayaka S., Brittian K.R., Long B.W., Higgins L.A., Bradley J.A., Audam T.N., Jurkovic A., Gumpert A.M., Harrison L.T., Hartyánszky I. (2020). Cardiomyocyte Oga Haploinsufficiency Increases O-GlcNAcylation but Hastens Ventricular Dysfunction Following Myocardial Infarction. PLoS ONE.

[105] Banerjee P.S., Ma J., Hart G.W. (2015). Diabetes-Associated Dysregulation of O-GlcNAcylation in Rat Cardiac Mitochondria. Proc. Natl. Acad. Sci. USA.

[106] Hu Y., Suarez J., Fricovsky E., Wang H., Scott B.T., Trauger S.A., Han W., Hu Y., Oyeleye M.O., Dillmann W.H. (2009). Increased Enzymatic O-GlcNAcylation of Mitochondrial Proteins Impairs Mitochondrial Function in Cardiac Myocytes Exposed to High Glucose. J. Biol. Chem..

[107] Prakoso D., Lim S.Y., Erickson J.R., Wallace R.S., Lees J.G., Tate M., Kiriazis H., Donner D.G., Henstridge D.C., Davey J.R. (2021). Fine-Tuning the Cardiac O-GlcNAcylation Regulatory Enzymes Governs the Functional and Structural Phenotype of the Diabetic Heart. Cardiovasc. Res..

[108] Ramirez-Correa G.A., Ma J., Slawson C., Zeidan Q., Lugo-Fagundo N.S., Xu M., Shen X., Gao W.D., Caceres V., Chakir K. (2015). Removal of Abnormal Myofilament O-GlcNAcylation Restores Ca^2+^ Sensitivity in Diabetic Cardiac Muscle. Diabetes.

[109] Lu S., Liao Z., Lu X., Katschinski D.M., Mercola M., Chen J., Brown J.H., Molkentin J.D., Bossuyt J., Bers D.M. (2020). Hyperglycemia Acutely Increases Cytosolic Reactive Oxygen Species via O-Linked GlcNAcylation and CaMKII Activation in Mouse Ventricular Myocytes. Circ. Res..

[110] Mesubi O.O., Rokita A.G., Abrol N., Wu Y., Chen B., Wang Q., Granger J.M., Tucker-Bartley A., Luczak E.D., Murphy K.R. (2021). Oxidized CaMKII and O-GlcNAcylation Cause Increased Atrial Fibrillation in Diabetic Mice by Distinct Mechanisms. J. Clin. Investig..

[111] Lunde I.G., Aronsen J.M., Kvaløy H., Qvigstad E., Sjaastad I., Tønnessen T., Christensen G., Grønning-Wang L.M., Carlson C.R. (2012). Cardiac O-GlcNAc Signaling Is Increased in Hypertrophy and Heart Failure. Physiol. Genom..

[112] Facundo H.T., Brainard R.E., Watson L.J., Ngoh G.A., Hamid T., Prabhu S.D., Jones S.P. (2012). O-GlcNAc Signaling Is Essential for NFAT-Mediated Transcriptional Reprogramming during Cardiomyocyte Hypertrophy. AJP Heart Circ. Physiol..

[113] Dassanayaka S., Brainard R.E., Watson L.J., Long B.W., Brittian K.R., DeMartino A.M., Aird A.L., Gumpert A.M., Audam T.N., Kilfoil P.J. (2017). Cardiomyocyte Ogt Limits Ventricular Dysfunction in Mice Following Pressure Overload without Affecting Hypertrophy. Basic Res. Cardiol..

[114] Ledee D., Smith L., Bruce M., Kajimoto M., Isern N., Portman M.A., Olson A.K. (2015). C-Myc Alters Substrate Utilization and O-GlcNAc Protein Posttranslational Modifications without Altering Cardiac Function during Early Aortic Constriction. PLoS ONE.

[115] Zhu W., El-Nachef D., Yang X., Ledee D., Olson A.K. (2019). O-GlcNAc Transferase Promotes Compensated Cardiac Function and Protein Kinase A O-GlcNAcylation During Early and Established Pathological Hypertrophy From Pressure Overload. J. Am. Heart Assoc..

[116] Gélinas R., Mailleux F., Dontaine J., Bultot L., Demeulder B., Ginion A., Daskalopoulos E.P., Esfahani H., Dubois-Deruy E., Lauzier B. (2018). AMPK Activation Counteracts Cardiac Hypertrophy by Reducing O-GlcNAcylation. Nat. Commun..

[117] Nabeebaccus A.A., Verma S., Zoccarato A., Emanuelli G., Santos C.X.C., Streckfuss-Bömeke K., Shah A.M. (2021). Cardiomyocyte Protein O-GlcNAcylation Is Regulated by GFAT1 Not GFAT2. Biochem. Biophys. Res. Commun..

[118] Tran D.H., May H.I., Li Q., Luo X., Huang J., Zhang G., Niewold E., Wang X., Gillette T.G., Deng Y. (2020). Chronic Activation of Hexosamine Biosynthesis in the Heart Triggers Pathological Cardiac Remodeling. Nat. Commun..

[119] Ishikita A., Matsushima S., Ikeda S., Okabe K., Nishimura R., Tadokoro T., Enzan N., Yamamoto T., Sada M., Tsutsui Y. (2021). GFAT2 Mediates Cardiac Hypertrophy through HBP-O-GlcNAcylation-Akt Pathway. Iscience.

[120] Brainard R.E., Facundo H.T. (2021). Cardiac Hypertrophy Drives PGC-1α Suppression Associated with Enhanced O-Glycosylation. Biochim. Biophys. Acta (BBA)-Mol. Basis Dis..

[121] Dubois-Deruy E., Belliard A., Mulder P., Bouvet M., Smet-Nocca C., Janel S., Lafont F., Beseme O., Amouyel P., Richard V. (2015). Interplay between Troponin T Phosphorylation and O-N-Acetylglucosaminylation in Ischaemic Heart Failure. Cardiovasc. Res..

[122] Laczy B., Marsh S.A., Brocks C.A., Wittmann I., Chatham J.C. (2010). Inhibition of O-GlcNAcase in Perfused Rat Hearts by NAG-Thiazolines at the Time of Reperfusion Is Cardioprotective in an O-GlcNAc-Dependent Manner. AJP Heart Circ. Physiol..

[123] Fülöp N., Zhang Z., Marchase R.B., Chatham J.C. (2007). Glucosamine Cardioprotection in Perfused Rat Hearts Associated with Increased O-Linked N-Acetylglucosamine Protein Modification and Altered P38 Activation. AJP Heart Circ. Physiol..

[124] Liu J., PANG Y., Chang T., Bounelis P., Chatham J., Marchase R. (2006). Increased Hexosamine Biosynthesis and Protein O-GlcNAc Levels Associated with Myocardial Protection against Calcium Paradox and Ischemia. J. Mol. Cell. Cardiol..

[125] Liu J., Marchase R.B., Chatham J.C. (2007). Glutamine-Induced Protection of Isolated Rat Heart from Ischemia/Reperfusion Injury Is Mediated via the Hexosamine Biosynthesis Pathway and Increased Protein O-GlcNAc Levels. J. Mol. Cell. Cardiol..

[126] Liu J., Marchase R.B., Chatham J.C. (2007). Increased O-GlcNAc Levels during Reperfusion Lead to Improved Functional Recovery and Reduced Calpain Proteolysis. Am. J. Physiol. Heart Circ. Physiol..

[127] Ngoh G.A., Watson L.J., Facundo H.T., Dillmann W., Jones S.P. (2008). Non-Canonical Glycosyltransferase Modulates Post-Hypoxic Cardiac Myocyte Death and Mitochondrial Permeability Transition. J. Mol. Cell. Cardiol..

[128] Giorgio V., von Stockum S., Antoniel M., Fabbro A., Fogolari F., Forte M., Glick G.D., Petronilli V., Zoratti M., Szabó I. (2013). Dimers of Mitochondrial ATP Synthase Form the Permeability Transition Pore. Proc. Natl. Acad. Sci. USA.

[129] Ngoh G.A., Hamid T., Prabhu S.D., Jones S.P. (2009). O-GlcNAc Signaling Attenuates ER Stress-Induced Cardiomyocyte Death. AJP Heart Circ. Physiol..

[130] Latorre-Muro P., O’Malley K.E., Bennett C.F., Perry E.A., Balsa E., Tavares C.D.J., Jedrychowski M., Gygi S.P., Puigserver P. (2021). A Cold-Stress-Inducible PERK/OGT Axis Controls TOM70-Assisted Mitochondrial Protein Import and Cristae Formation. Cell Metab..

[131] Zeidan Q., Wang Z., Maio A.D., Hart G.W. (2010). O-GlcNAc Cycling Enzymes Associate with the Translational Machinery and Modify Core Ribosomal Proteins. Mol. Biol. Cell.

[132] Li X., Zhu Q., Shi X., Cheng Y., Li X., Xu H., Duan X., Hsieh-Wilson L.C., Chu J., Pelletier J. (2019). O-GlcNAcylation of Core Components of the Translation Initiation Machinery Regulates Protein Synthesis. Proc. Natl. Acad. Sci. USA.

[133] Jang I., Kim H.B., Seo H., Kim J.Y., Choi H., Yoo J.S., Kim J., Cho J.W. (2015). O-GlcNAcylation of EIF2α Regulates the Phospho-EIF2α-Mediated ER Stress Response. Biochim. Biophys. Acta.

[134] Lim K.-H., Chang H.-I. (2006). O-Linked N-Acetylglucosamine Suppresses Thermal Aggregation of Sp1. FEBS Lett..

[135] Zhang X., Shu X.E., Qian S.-B. (2018). O-GlcNAc Modification of EIF4GI Acts as a Translational Switch in Heat Shock Response. Nat. Publ. Group.

[136] Alejandro E.U., Bozadjieva N., Kumusoglu D., Abdulhamid S., Levine H., Haataja L., Vadrevu S., Satin L.S., Arvan P., Bernal-Mizrachi E. (2015). Disruption of O-Linked N-Acetylglucosamine Signaling Induces ER Stress and β Cell Failure. Cell Rep..

[137] Zou L., Collins H.E., Young M.E., Zhang J., Wende A.R., Darley-Usmar V.M., Chatham J.C. (2021). The Identification of a Novel Calcium-Dependent Link Between NAD+ and Glucose Deprivation-Induced Increases in Protein O-GlcNAcylation and ER Stress. Front. Mol. Biosci..

[138] Taylor R.P., Parker G.J., Hazel M.W., Soesanto Y., Fuller W., Yazzie M.J., McClain D.A. (2008). Glucose Deprivation Stimulates O-GlcNAc Modification of Proteins through Up-Regulation of O-Linked N-Acetylglucosaminyltransferase*. J. Biol. Chem..

[139] Miller W.P., Mihailescu M.L., Yang C., Barber A.J., Kimball S.R., Jefferson L.S., Dennis M.D. (2016). The Translational Repressor 4E-BP1 Contributes to Diabetes-Induced Visual Dysfunction. Investig. Opthalmology Vis. Sci..

[140] Dennis M.D., Shenberger J.S., Stanley B.A., Kimball S.R., Jefferson L.S. (2013). Hyperglycemia Mediates a Shift from Cap-Dependent to Cap-Independent Translation Via a 4E-BP1–Dependent Mechanism. Diabetes.

[141] Dierschke S.K., Miller W.P., Favate J.S., Shah P., Kawasawa Y.I., Salzberg A.C., Kimball S.R., Jefferson L.S., Dennis M.D. (2019). O-GlcNAcylation Alters the Selection of MRNAs for Translation and Promotes 4E-BP1–Dependent Mitochondrial Dysfunction in the Retina. J. Biol. Chem..

[142] Yang S., Zou L.-Y., Bounelis P., Chaudry I., Chatham J.C., Marchase R.B. (2006). Glucosamine Administration during Resuscitation Improves Organ Function after Trauma Hemorrhage. Shock.

[143] Nöt L.G., Marchase R.B., Fülöp N., Brocks C.A., Chatham J.C. (2007). Glucosamine Administration Improves Survival Rate after Severe Hemorrhagic Shock Combined With Trauma in Rats. Shock.

[144] Zou L., Yang S., Champattanachai V., Hu S., Chaudry I.H., Marchase R.B., Chatham J.C. (2008). Glucosamine Improves Cardiac Function Following Trauma-Hemorrhage by Increased Protein O-GlcNAcylation and Attenuation of NF- B Signaling. AJP Heart Circ. Physiol..

[145] Ramakrishnan P., Clark P.M., Mason D.E., Peters E.C., Hsieh-Wilson L.C., Baltimore D. (2013). Activation of the Transcriptional Function of the NF-ΚB Protein c-Rel by O-GlcNAc Glycosylation. Sci. Signal..

[146] Ma Z., Chalkley R.J., Vosseller K. (2017). Hyper-O-GlcNAcylation Activates Nuclear Factor κ-Light-Chain-Enhancer of Activated B Cells (NF-ΚB) Signaling through Interplay with Phosphorylation and Acetylation. J. Biological. Chem..

[147] Nöt L.G., Brocks C.A., Vámhidy L., Marchase R.B., Chatham J.C. (2010). Increased O-Linked β-N-Acetylglucosamine Levels on Proteins Improves Survival, Reduces Inflammation and Organ Damage 24 Hours after Trauma-Hemorrhage in Rats. Crit. Care Med..

[148] Ferron M., Cadiet J., Persello A., Prat V., Denis M., Erraud A., Aillerie V., Mevel M., Bigot E., Chatham J.C. (2019). O-GlcNAc Stimulation: A New Metabolic Approach to Treat Septic Shock. Sci. Rep..

[149] Denis M., Dupas T., Persello A., Dontaine J., Bultot L., Betus C., Pelé T., Dhot J., Erraud A., Maillard A. (2021). An O-GlcNAcylomic Approach Reveals ACLY as a Potential Target in Sepsis in the Young Rat. Int. J. Mol. Sci..

[150] Dupas T., Persello A., Blangy-Letheule A., Denis M., Erraud A., Aillerie V., Leroux A.A., Rivière M., Lebreton J., Tessier A. (2022). Beneficial Effects of O-GlcNAc Stimulation in a Young Rat Model of Sepsis: Beyond Modulation of Gene Expression. Int. J. Mol. Sci..

[151] Liao L., Cheng D., Wang J., Duong D.M., Losik T.G., Gearing M., Rees H.D., Lah J.J., Levey A.I., Peng J. (2004). Proteomic Characterization of Postmortem Amyloid Plaques Isolated by Laser Capture Microdissection*. J. Biol. Chem..

[152] Woerner A.C., Frottin F., Hornburg D., Feng L.R., Meissner F., Patra M., Tatzelt J., Mann M., Winklhofer K.F., Hartl F.U. (2016). Cytoplasmic Protein Aggregates Interfere with Nucleocytoplasmic Transport of Protein and RNA. Science.

[153] Riemenschneider H., Guo Q., Bader J., Frottin F., Farny D., Kleinberger G., Haass C., Mann M., Hartl F.U., Baumeister W. (2022). Gel-like Inclusions of C-terminal Fragments of TDP-43 Sequester Stalled Proteasomes in Neurons. EMBO Rep..

[154] Wang A.C., Jensen E.H., Rexach J.E., Vinters H.V., Hsieh-Wilson L.C. (2016). Loss of O-GlcNAc Glycosylation in Forebrain Excitatory Neurons Induces Neurodegeneration. Proc. Natl. Acad. Sci. USA.

[155] Park J., Ha H.-J., Chung E.S., Baek S.H., Cho Y., Kim H.K., Han J., Sul J.H., Lee J., Kim E. (2021). O-GlcNAcylation Ameliorates the Pathological Manifestations of Alzheimer’s Disease by Inhibiting Necroptosis. Sci. Adv..

[156] Yao P.J., Coleman P.D. (1998). Reduction of O-Linked N-Acetylglucosamine-Modified Assembly Protein-3 in Alzheimer’s Disease. J. Neurosci. Off. J. Soc. Neurosci..

[157] Liu F., Iqbal K., Grundke-Iqbal I., Hart G.W., Gong C.-X. (2004). O-GlcNAcylation Regulates Phosphorylation of Tau: A Mechanism Involved in Alzheimer’s Disease. Proc. Natl. Acad. Sci. USA.

[158] Wang S., Yang F., Petyuk V.A., Shukla A.K., Monroe M.E., Gritsenko M.A., Rodland K.D., Smith R.D., Qian W.-J., Gong C.-X. (2017). Quantitative Proteomics Identifies Altered O-GlcNAcylation of Structural, Synaptic and Memory-Associated Proteins in Alzheimer’s Disease. J. Pathol..

[159] Arnold C.S., Johnson G.V., Cole R.N., Dong D.L., Lee M., Hart G.W. (1996). The Microtubule-Associated Protein Tau Is Extensively Modified with O-Linked N-Acetylglucosamine. J. Biol. Chem..

[160] Liu F., Shi J., Tanimukai H., Gu J., Gu J., Grundke-Iqbal I., Iqbal K., Gong C.-X. (2009). Reduced O-GlcNAcylation Links Lower Brain Glucose Metabolism and Tau Pathology in Alzheimer’s Disease. Brain.

[161] Yuzwa S.A., Yadav A.K., Skorobogatko Y., Clark T., Vosseller K., Vocadlo D.J. (2011). Mapping O-GlcNAc Modification Sites on Tau and Generation of a Site-Specific O-GlcNAc Tau Antibody. Amino Acids.

[162] Yuzwa S.A., Shan X., Macauley M.S., Clark T., Skorobogatko Y., Vosseller K., Vocadlo D.J. (2012). Increasing O-GlcNAc Slows Neurodegeneration and Stabilizes Tau against Aggregation. Nat. Chem. Biol..

[163] Yuzwa S.A., Cheung A.H., Okon M., McIntosh L.P., Vocadlo D.J. (2014). O-GlcNAc Modification of Tau Directly Inhibits Its Aggregation without Perturbing the Conformational Properties of Tau Monomers. J. Mol. Biol..

[164] Cantrelle F.-X., Loyens A., Trivelli X., Reimann O., Despres C., Gandhi N.S., Hackenberger C.P.R., Landrieu I., Smet-Nocca C. (2021). Phosphorylation and O-GlcNAcylation of the PHF-1 Epitope of Tau Protein Induce Local Conformational Changes of the C-Terminus and Modulate Tau Self-Assembly Into Fibrillar Aggregates. Front. Mol. Neurosci..

[165] Borghgraef P., Menuet C., Theunis C., Louis J.V., Devijver H., Maurin H., Smet-Nocca C., Lippens G., Hilaire G., Gijsen H. (2013). Increasing Brain Protein O-GlcNAc-Ylation Mitigates Breathing Defects and Mortality of Tau.P301L Mice. PLoS ONE.

[166] Hastings N.B., Wang X., Song L., Butts B.D., Grotz D., Hargreaves R., Hess J.F., Hong K.-L.K., Huang C.R.-R., Hyde L. (2017). Inhibition of O-GlcNAcase Leads to Elevation of O-GlcNAc Tau and Reduction of Tauopathy and Cerebrospinal Fluid Tau in RTg4510 Mice. Mol. Neurodegener..

[167] Wang X., Li W., Marcus J., Pearson M., Song L., Smith K., Terracina G., Lee J., Hong K.-L.K., Lu S.X. (2020). MK-8719, a Novel and Selective O-GlcNAcase Inhibitor That Reduces the Formation of Pathological Tau and Ameliorates Neurodegeneration in a Mouse Model of Tauopathy. J. Pharmacol. Exp. Ther..

[168] Alquezar C., Arya S., Kao A.W. (2021). Tau Post-Translational Modifications: Dynamic Transformers of Tau Function, Degradation, and Aggregation. Front. Neurol..

[169] Yuzwa S.A., Macauley M.S., Heinonen J.E., Shan X., Dennis R.J., He Y., Whitworth G.E., Stubbs K.A., McEachern E.J., Davies G.J. (2008). A Potent Mechanism-Inspired O-GlcNAcase Inhibitor That Blocks Phosphorylation of Tau in Vivo. Nat. Chem. Biol..

[170] Graham D.L., Gray A.J., Joyce J.A., Yu D., O’Moore J., Carlson G.A., Shearman M.S., Dellovade T.L., Hering H. (2014). Increased O-GlcNAcylation Reduces Pathological Tau without Affecting Its Normal Phosphorylation in a Mouse Model of Tauopathy. Neuropharmacology.

[171] Jiménez J.S. Macromolecular Structures and Proteins Interacting with the Microtubule Associated Tau Protein. Neuroscience.

[172] Tracy T.E., Madero-Pérez J., Swaney D.L., Chang T.S., Moritz M., Konrad C., Ward M.E., Stevenson E., Hüttenhain R., Kauwe G. (2022). Tau Interactome Maps Synaptic and Mitochondrial Processes Associated with Neurodegeneration. Cell.

[173] Sui D., Liu M., Kuo M.-H. (2015). In Vitro Aggregation Assays Using Hyperphosphorylated Tau Protein. J. Vis. Exp..

[174] Gorantla N.V., Chinnathambi S. (2021). Autophagic Pathways to Clear the Tau Aggregates in Alzheimer’s Disease. Cell Mol. Neurobiol..

[175] Caballero B., Bourdenx M., Luengo E., Diaz A., Sohn P.D., Chen X., Wang C., Juste Y.R., Wegmann S., Patel B. (2021). Acetylated Tau Inhibits Chaperone-Mediated Autophagy and Promotes Tau Pathology Propagation in Mice. Nat. Commun..

[176] Guo B., Liang Q., Li L., Hu Z., Wu F., Zhang P., Ma Y., Zhao B., Kovács A.L., Zhang Z. (2014). O-GlcNAc-Modification of SNAP-29 Regulates Autophagosome Maturation. Nat. Cell Biol..

[177] Ruan H.-B., Ma Y., Torres S., Zhang B., Feriod C., Heck R.M., Qian K., Fu M., Li X., Nathanson M.H. (2017). Calcium-Dependent O-GlcNAc Signaling Drives Liver Autophagy in Adaptation to Starvation. Genes Dev..

[178] Luu L., Ciccotosto G.D., Cappai R. (2021). The Alzheimer’s Disease Amyloid Precursor Protein and Its Neuritogenic Actions. Curr. Alzheimer Res..

[179] Higgins L.S., Murphy G.M., Forno L.S., Catalano R., Cordell B. (1996). P3 Beta-Amyloid Peptide Has a Unique and Potentially Pathogenic Immunohistochemical Profile in Alzheimer’s Disease Brain. Am. J. Pathol..

[180] Kuhn A.J., Abrams B.S., Knowlton S., Raskatov J.A. (2020). Alzheimer’s Disease “Non-Amyloidogenic” P3 Peptide Revisited: A Case for Amyloid-α. ACS Chem. Neurosci..

[181] Yuzwa S.A., Shan X., Jones B.A., Zhao G., Woodward M.L., Li X., Zhu Y., McEachern E.J., Silverman M.A., Watson N.V. (2014). Pharmacological Inhibition of O-GlcNAcase (OGA) Prevents Cognitive Decline and Amyloid Plaque Formation in Bigenic Tau/APP Mutant Mice. Mol. Neurodegener..

[182] Kim C., Nam D.W., Park S.Y., Song H., Hong H.S., Boo J.H., Jung E.S., Kim Y., Baek J.Y., Kim K.S. (2013). O-Linked β-N-Acetylglucosaminidase Inhibitor Attenuates β-Amyloid Plaque and Rescues Memory Impairment. Neurobiol. Aging.

[183] Jacobsen K.T., Iverfeldt K. (2011). O-GlcNAcylation Increases Non-Amyloidogenic Processing of the Amyloid-β Precursor Protein (APP). Biochem. Biophys. Res. Commun..

[184] Chun Y.S., Kwon O.-H., Chung S. (2017). O-GlcNAcylation of Amyloid-β Precursor Protein at Threonine 576 Residue Regulates Trafficking and Processing. Biochem. Biophys. Res. Commun..

[185] Chun Y.S., Park Y., Oh H.G., Kim T.-W., Yang H.O., Park M.K., Chung S. (2015). O-GlcNAcylation Promotes Non-Amyloidogenic Processing of Amyloid-β Protein Precursor via Inhibition of Endocytosis from the Plasma Membrane. J. Alzheimer’s Dis..

[186] Griffith L.S., Mathes M., Schmitz B. (1995). Beta-Amyloid Precursor Protein Is Modified with O-Linked N-Acetylglucosamine. J. Neurosci. Res..

[187] Runwal G., Edwards R.H. (2021). The Membrane Interactions of Synuclein: Physiology and Pathology. Annu. Rev. Pathol. Mech. Dis..

[188] de Boni L., Watson A.H., Zaccagnini L., Wallis A., Zhelcheska K., Kim N., Sanderson J., Jiang H., Martin E., Cantlon A. (2022). Brain Region-Specific Susceptibility of Lewy Body Pathology in Synucleinopathies Is Governed by α-Synuclein Conformations. Acta Neuropathol..

[189] Dettmer U., Newman A.J., von Saucken V.E., Bartels T., Selkoe D. (2015). KTKEGV Repeat Motifs Are Key Mediators of Normal α-Synuclein Tetramerization: Their Mutation Causes Excess Monomers and Neurotoxicity. Proc. Natl. Acad. Sci. USA.

[190] Imberdis T., Fanning S., Newman A., Ramalingam N., Dettmer U. (2019). Alpha-Synuclein, Methods and Protocols. Methods Mol. Biol..

[191] Benskey M.J., Perez R.G., Manfredsson F.P. (2016). The Contribution of Alpha Synuclein to Neuronal Survival and Function—Implications for Parkinson’s Disease. J. Neurochem..

[192] Yoo H., Lee J., Kim B., Moon H., Jeong H., Lee K., Song W.J., Hur J.K., Oh Y. (2022). Role of Post-Translational Modifications on the Alpha-Synuclein Aggregation-Related Pathogenesis of Parkinson’s Disease. BMB Rep..

[193] Balana A.T., Pratt M.R. (2021). Mechanistic Roles for Altered O-GlcNAcylation in Neurodegenerative Disorders. Biochem. J..

[194] Wani W.Y., Ouyang X., Benavides G.A., Redmann M., Cofield S.S., Shacka J.J., Chatham J.C., Darley-Usmar V., Zhang J. (2017). O-GlcNAc Regulation of Autophagy and α-Synuclein Homeostasis; Implications for Parkinson’s Disease. Mol. Brain.

[195] Marotta N.P., Lin Y.H., Lewis Y.E., Ambroso M.R., Zaro B.W., Roth M.T., Arnold D.B., Langen R., Pratt M.R. (2015). O-GlcNAc Modification Blocks the Aggregation and Toxicity of the Protein α-Synuclein Associated with Parkinson’s Disease. Nat. Chem..

[196] Levine P.M., Galesic A., Balana A.T., Mahul-Mellier A.-L., Navarro M.X., Leon C.A.D., Lashuel H.A., Pratt M.R. (2019). α-Synuclein O-GlcNAcylation Alters Aggregation and Toxicity, Revealing Certain Residues as Potential Inhibitors of Parkinson’s Disease. Proc. Natl. Acad. Sci. USA.

[197] Lewis Y.E., Galesic A., Levine P.M., Leon C.A.D., Lamiri N., Brennan C.K., Pratt M.R. (2017). O-GlcNAcylation of α-Synuclein at Serine 87 Reduces Aggregation without Affecting Membrane Binding. ACS Chem. Biol..

[198] Tavassoly O., Yue J., Vocadlo D.J. (2021). Pharmacological Inhibition and Knockdown of O-GlcNAcase Reduces Cellular Internalization of A-synuclein Preformed Fibrils. FEBS J..

[199] Ihse E., Yamakado H., van Wijk X.M., Lawrence R., Esko J.D., Masliah E. (2017). Cellular Internalization of Alpha-Synuclein Aggregates by Cell Surface Heparan Sulfate Depends on Aggregate Conformation and Cell Type. Sci. Rep..

[200] Aulić S., Masperone L., Narkiewicz J., Isopi E., Bistaffa E., Ambrosetti E., Pastore B., Cecco E.D., Scaini D., Zago P. (2017). α-Synuclein Amyloids Hijack Prion Protein to Gain Cell Entry, Facilitate Cell-to-Cell Spreading and Block Prion Replication. Sci. Rep..

[201] Zhang S., Liu Y.-Q., Jia C., Lim Y.-J., Feng G., Xu E., Long H., Kimura Y., Tao Y., Zhao C. (2021). Mechanistic Basis for Receptor-Mediated Pathological α-Synuclein Fibril Cell-to-Cell Transmission in Parkinson’s Disease. Proc. Natl. Acad. Sci. USA.

[202] Scarlino S., Domi T., Pozzi L., Romano A., Pipitone G.B., Falzone Y.M., Mosca L., Penco S., Lunetta C., Sansone V. (2020). Burden of Rare Variants in ALS and Axonal Hereditary Neuropathy Genes Influence Survival in ALS: Insights from a Next Generation Sequencing Study of an Italian ALS Cohort. Int. J. Mol. Sci..

[203] Kim G., Gautier O., Tassoni-Tsuchida E., Ma X.R., Gitler A.D. (2020). ALS Genetics: Gains, Losses, and Implications for Future Therapies. Neuron.

[204] Gasset-Rosa F., Lu S., Yu H., Chen C., Melamed Z., Guo L., Shorter J., Cruz S.D., Cleveland D.W. (2019). Cytoplasmic TDP-43 De-Mixing Independent of Stress Granules Drives Inhibition of Nuclear Import, Loss of Nuclear TDP-43, and Cell Death. Neuron.

[205] Maraschi A., Gumina V., Dragotto J., Colombrita C., Mompeán M., Buratti E., Silani V., Feligioni M., Ratti A. (2021). SUMOylation Regulates TDP-43 Splicing Activity and Nucleocytoplasmic Distribution. Mol. Neurobiol..

[206] Buratti E. (2018). TDP-43 Post-Translational Modifications in Health and Disease. Expert Opin. Ther. Targets.

[207] da Silva L.A.G., Simonetti F., Hutten S., Riemenschneider H., Sternburg E.L., Pietrek L.M., Gebel J., Dötsch V., Edbauer D., Hummer G. (2022). Disease-linked TDP-43 Hyperphosphorylation Suppresses TDP-43 Condensation and Aggregation. EMBO J..

[208] Zhao M., Yao X., Wei P., Zhao C., Cheng M., Zhang D., Xue W., He W., Xue W., Zuo X. (2021). O-GlcNAcylation of TDP-43 Suppresses Proteinopathies and Promotes TDP-43’s MRNA Splicing Activity. EMBO Rep..

[209] Cunha-Oliveira T., Montezinho L., Mendes C., Firuzi O., Saso L., Oliveira P.J., Silva F.S.G. (2020). Oxidative Stress in Amyotrophic Lateral Sclerosis: Pathophysiology and Opportunities for Pharmacological Intervention. Oxidative Med. Cell. Longev..

[210] Ratti A., Gumina V., Lenzi P., Bossolasco P., Fulceri F., Volpe C., Bardelli D., Pregnolato F., Maraschi A., Fornai F. (2020). Chronic Stress Induces Formation of Stress Granules and Pathological TDP-43 Aggregates in Human ALS Fibroblasts and IPSC-Motoneurons. Neurobiol. Dis..

[211] Zuo X., Zhou J., Li Y., Wu K., Chen Z., Luo Z., Zhang X., Liang Y., Esteban M.A., Zhou Y. (2021). TDP-43 Aggregation Induced by Oxidative Stress Causes Global Mitochondrial Imbalance in ALS. Nat. Struct. Mol. Biol..

[212] Romano N., Catalani A., Lattante S., Belardo A., Proietti S., Bertini L., Silvestri F., Catalani E., Cervia D., Zolla L. (2020). ALS Skin Fibroblasts Reveal Oxidative Stress and ERK1/2-Mediated Cytoplasmic Localization of TDP-43. Cell Signal..

[213] Riancho J., Castanedo-Vázquez D., Gil-Bea F., Tapia O., Arozamena J., Durán-Vían C., Sedano M.J., Berciano M.T., de Munain A.L., Lafarga M. (2020). ALS-Derived Fibroblasts Exhibit Reduced Proliferation Rate, Cytoplasmic TDP-43 Aggregation and a Higher Susceptibility to DNA Damage. J. Neurol..

[214] Shan X., Vocadlo D.J., Krieger C. (2012). Reduced Protein O-Glycosylation in the Nervous System of the Mutant SOD1 Transgenic Mouse Model of Amyotrophic Lateral Sclerosis. Neurosci. Lett..

[215] Hsieh Y.-L., Su F.-Y., Tsai L.-K., Huang C.-C., Ko Y.-L., Su L.-W., Chen K.-Y., Shih H.-M., Hu C.-M., Lee W.-H. (2019). NPGPx-Mediated Adaptation to Oxidative Stress Protects Motor Neurons from Degeneration in Aging by Directly Modulating O-GlcNAcase. Cell Rep..

[216] Comer F.I., Vosseller K., Wells L., Accavitti M.A., Hart G.W. (2001). Characterization of a Mouse Monoclonal Antibody Specific for O-Linked N-Acetylglucosamine. Anal. Biochem..

[217] Teo C.F., Ingale S., Wolfert M.A., Elsayed G.A., Nöt L.G., Chatham J.C., Wells L., Boons G.-J. (2010). Glycopeptide-Specific Monoclonal Antibodies Suggest New Roles for O-GlcNAc. Nat. Chem. Biol..

[218] Liu Y., Chen Q., Zhang N., Zhang K., Dou T., Cao Y., Liu Y., Li K., Hao X., Xie X. (2020). Proteomic Profiling and Genome-Wide Mapping of O-GlcNAc Chromatin-Associated Proteins Reveal an O-GlcNAc-Regulated Genotoxic Stress Response. Nat. Commun..

[219] Zachara N.E., Molina H., Wong K.Y., Pandey A., Hart G.W. (2010). The Dynamic Stress-Induced “O-GlcNAc-Ome” Highlights Functions for O-GlcNAc in Regulating DNA Damage/Repair and Other Cellular Pathways. Amino Acids.

[220] Heap G.A., Heel D.A. (2009). van The Genetics of Chronic Inflammatory Diseases. Hum. Mol. Genet..

[221] Hotamisligil G.S. (2006). Inflammation and Metabolic Disorders. Nature.

[222] Schmid-Schönbein G.W. (2006). Analysis of Inflammation. Biomed. Eng..

[223] Xu Y.-R., Lei C.-Q. (2021). TAK1-TABs Complex: A Central Signalosome in Inflammatory Responses. Front. Immunol..

[224] Shinohara H., Yasuda T., Kurosaki T. (2016). TAK1 Adaptor Proteins, TAB2 and TAB3, Link the Signalosome to B-cell Receptor-induced IKK Activation. FEBS Lett..

[225] Ninomiya-Tsuji J., Kishimoto K., Hiyama A., Inoue J., Cao Z., Matsumoto K. (1999). The Kinase TAK1 Can Activate the NIK-IκB as Well as the MAP Kinase Cascade in the IL-1 Signalling Pathway. Nature.

[226] Fan Y., Yu Y., Shi Y., Sun W., Xie M., Ge N., Mao R., Chang A., Xu G., Schneider M.D. (2010). Lysine 63-Linked Polyubiquitination of TAK1 at Lysine 158 Is Required for Tumor Necrosis Factor α- and Interleukin-1β-Induced IKK/NF-ΚB and JNK/AP-1 Activation*. J. Biol. Chem..

[227] Tao T., He Z., Shao Z., Lu H. (2016). TAB3 O-GlcNAcylation Promotes Metastasis of Triple Negative Breast Cancer. Oncotarget.

[228] Woo C.M., Lund P.J., Huang A.C., Davis M.M., Bertozzi C.R., Pitteri S.J. (2018). Mapping and Quantification of Over 2000 O-Linked Glycopeptides in Activated Human T Cells with Isotope-Targeted Glycoproteomics (Isotag). Mol. Cell Proteom..

[229] Yang W.H., Park S.Y., Nam H.W., Kim D.H., Kang J.G., Kang E.S., Kim Y.S., Lee H.C., Kim K.S., Cho J.W. (2008). NFκB Activation Is Associated with Its O-GlcNAcylation State under Hyperglycemic Conditions. Proc. Natl. Acad. Sci. USA.

[230] Kawauchi K., Araki K., Tobiume K., Tanaka N. (2009). Loss of P53 Enhances Catalytic Activity of IKKβ through O-Linked β-N-Acetyl Glucosamine Modification. Proc. Natl. Acad. Sci. USA.

[231] Higashimoto T., Chan N., Lee Y.-K., Zandi E. (2008). Regulation of IκB Kinase Complex by Phosphorylation of γ-Binding Domain of IκB Kinase β by Polo-like Kinase 1*. J. Biol. Chem..

[232] Schomer-Miller B., Higashimoto T., Lee Y.-K., Zandi E. (2006). Regulation of IκB Kinase (IKK) Complex by IKKγ-Dependent Phosphorylation of the T-Loop and C Terminus of IKKβ*. J. Biol. Chem..

[233] Kneass Z.T., Marchase R.B. (2004). Neutrophils Exhibit Rapid Agonist-Induced Increases in Protein-Associated O-GlcNAc*. J. Biol. Chem..

[234] Kneass Z.T., Marchase R.B. (2005). Protein O-GlcNAc Modulates Motility-Associated Signaling Intermediates in Neutrophils*. J. Biol. Chem..

[235] Li X., Gong W., Wang H., Li T., Attri K.S., Lewis R.E., Kalil A.C., Bhinderwala F., Powers R., Yin G. (2019). O-GlcNAc Transferase Suppresses Inflammation and Necroptosis by Targeting Receptor-Interacting Serine/Threonine-Protein Kinase 3. Immunity.

[236] Speir M., Lawlor K.E. (2020). RIP-Roaring Inflammation: RIPK1 and RIPK3 Driven NLRP3 Inflammasome Activation and Autoinflammatory Disease. Semin. Cell Dev. Biol..

[237] Yang Y., Wang H., Kouadir M., Song H., Shi F. (2019). Recent Advances in the Mechanisms of NLRP3 Inflammasome Activation and Its Inhibitors. Cell Death Dis..

[238] Li T., Li X., Attri K.S., Liu C., Li L., Herring L.E., Asara J.M., Lei Y.L., Singh P.K., Gao C. (2018). O-GlcNAc Transferase Links Glucose Metabolism to MAVS-Mediated Antiviral Innate Immunity. Cell Host Microbe.

[239] Shikhman A.R., Kuhn K., Alaaeddine N., Lotz M. (2001). N-Acetylglucosamine Prevents IL-1β-Mediated Activation of Human Chondrocytes. J. Immunol..

[240] Largo R., Alvarez-Soria M.A., Díez-Ortego I., Calvo E., Sánchez-Pernaute O., Egido J., Herrero-Beaumont G. (2003). Glucosamine Inhibits IL-1β-Induced NFκB Activation in Human Osteoarthritic Chondrocytes. Osteoarthr. Cartil..

[241] d’Abusco A.S., Calamia V., Cicione C., Grigolo B., Politi L., Scandurra R. (2007). Glucosamine Affects Intracellular Signalling through Inhibition of Mitogen-Activated Protein Kinase Phosphorylation in Human Chondrocytes. Arthritis Res. Ther..

[242] Hwang Y.P., Kim H.G., Han E.H., Choi J.H., Park B.H., Jung K.H., Shin Y.C., Jeong H.G. (2011). N-Acetylglucosamine Suppress Collagenases Activation in Ultraviolet B-Irradiated Human Dermal Fibroblasts: Involvement of Calcium Ions and Mitogen-Activated Protein Kinases. J. Dermatol. Sci..

[243] Li X., Zhang Z., Li L., Gong W., Lazenby A.J., Swanson B.J., Herring L.E., Asara J.M., Singer J.D., Wen H. (2017). Myeloid-Derived Cullin 3 Promotes STAT3 Phosphorylation by Inhibiting OGT Expression and Protects against Intestinal Inflammation. J. Exp. Med..

[244] Kessenbrock K., Plaks V., Werb Z. (2010). Matrix Metalloproteinases: Regulators of the Tumor Microenvironment. Cell.

[245] Kandhwal M., Behl T., Singh S., Sharma N., Arora S., Bhatia S., Al-Harrasi A., Sachdeva M., Bungau S. (2021). Role of Matrix Metalloproteinase in Wound Healing. Am. J. Transl. Res..

[246] Gorter R., Baron W. (2020). Matrix Metalloproteinases Shape the Oligodendrocyte (Niche) during Development and upon Demyelination. Neurosci. Lett..

[247] Huang J.-B., Clark A.J., Petty H.R. (2007). The Hexosamine Biosynthesis Pathway Negatively Regulates IL-2 Production by Jurkat T Cells. Cell Immunol..

[248] Xing D., Gong K., Feng W., Nozell S.E., Chen Y.-F., Chatham J.C., Oparil S. (2011). O-GlcNAc Modification of NFκB P65 Inhibits TNF-α-Induced Inflammatory Mediator Expression in Rat Aortic Smooth Muscle Cells. PLoS ONE.

[249] Someya A., Ikegami T., Sakamoto K., Nagaoka I. (2016). Glucosamine Downregulates the IL-1β-Induced Expression of Proinflammatory Cytokine Genes in Human Synovial MH7A Cells by O-GlcNAc Modification-Dependent and -Independent Mechanisms. PLoS ONE.

[250] Ortiz-Meoz R.F., Jiang J., Lazarus M.B., Orman M., Janetzko J., Fan C., Duveau D.Y., Tan Z.-W., Thomas C.J., Walker S. (2015). A Small Molecule That Inhibits OGT Activity in Cells. ACS Chem. Biol..

[251] Zebrucka K.P., Koryga I., Mnich K., Ljujic M., Samali A., Gorman A.M. (2016). The Integrated Stress Response. EMBO Rep..

[252] Mukherjee T., Ramaglia V., Abdel-Nour M., Bianchi A.A., Tsalikis J., Chau H.N., Kalia S.K., Kalia L.V., Chen J.-J., Arnoult D. (2021). The EIF2α Kinase HRI Triggers the Autophagic Clearance of Cytosolic Protein Aggregates. J. Biol. Chem..

[253] Donnelly N., Gorman A.M., Gupta S., Samali A. (2012). The EIF2α Kinases: Their Structures and Functions. Cell Mol. Life Sci..

[254] Dar A.C., Dever T.E., Sicheri F. (2005). Higher-Order Substrate Recognition of EIF2α by the RNA-Dependent Protein Kinase PKR. Cell.

[255] Zhang P., McGrath B.C., Reinert J., Olsen D.S., Lei L., Gill S., Wek S.A., Vattem K.M., Wek R.C., Kimball S.R. (2002). The GCN2 EIF2alpha Kinase Is Required for Adaptation to Amino Acid Deprivation in Mice. Mol. Cell Biol..

[256] Harding H.P., Novoa I., Zhang Y., Zeng H., Wek R., Schapira M., Ron D. (2000). Regulated Translation Initiation Controls Stress-Induced Gene Expression in Mammalian Cells. Mol. Cell.

[257] Vattem K.M., Wek R.C. (2004). Reinitiation Involving Upstream ORFs Regulates ATF4 MRNA Translation in Mammalian Cells. Proc. Natl. Acad. Sci. USA.

[258] Palam L.R., Baird T.D., Wek R.C. (2011). Phosphorylation of EIF2 Facilitates Ribosomal Bypass of an Inhibitory Upstream ORF to Enhance CHOP Translation. J. Biol. Chem..

[259] Jiang H.-Y., Wek S.A., McGrath B.C., Lu D., Hai T., Harding H.P., Wang X., Ron D., Cavener D.R., Wek R.C. (2004). Activating Transcription Factor 3 Is Integral to the Eukaryotic Initiation Factor 2 Kinase Stress Response. Mol. Cell Biol..

[260] Kaspar S., Oertlin C., Szczepanowska K., Kukat A., Senft K., Lucas C., Brodesser S., Hatzoglou M., Larsson O., Topisirovic I. (2020). Adaptation to Mitochondrial Stress Requires CHOP-Directed Tuning of ISR. Sci. Adv..

[261] Dey S., Baird T.D., Zhou D., Palam L.R., Spandau D.F., Wek R.C. (2010). Both Transcriptional Regulation and Translational Control of ATF4 Are Central to the Integrated Stress Response. J. Biol. Chem..

[262] Jiang H.-Y., Jiang L., Wek R.C. (2007). The Eukaryotic Initiation Factor-2 Kinase Pathway Facilitates Differential GADD45a Expression in Response to Environmental Stress*. J. Biol. Chem..

[263] Luo S., Baumeister P., Yang S., Abcouwer S.F., Lee A.S. (2003). Induction of Grp78/BiP by Translational Block: Activation of the Grp78 Promoter by ATF4 through and Upstream ATF/CRE Site Independent of the Endoplasmic Reticulum Stress Elements. J. Biological. Chem..

[264] Teske B.F., Fusakio M.E., Zhou D., Shan J., McClintick J.N., Kilberg M.S., Wek R.C. (2013). CHOP Induces Activating Transcription Factor 5 (ATF5) to Trigger Apoptosis in Response to Perturbations in Protein Homeostasis. Mol. Biol. Cell.

[265] Hahne H., Gholami A.M., Kuster B. (2012). Discovery of O-GlcNAc-Modified Proteins in Published Large-Scale Proteome Data. Mol. Cell. Proteom..

[266] Zhang N., Zhu T., Yu K., Shi M., Wang X., Wang L., Huang T., Li W., Liu Y., Zhang J. (2019). Elevation of O-GlcNAc and GFAT Expression by Nicotine Exposure Promotes Epithelial-mesenchymal Transition and Invasion in Breast Cancer Cells. Cell Death Dis..

[267] Chaveroux C., Sarcinelli C., Barbet V., Belfeki S., Barthelaix A., Ferraro-Peyret C., Lebecque S., Renno T., Bruhat A., Fafournoux P. (2016). Nutrient Shortage Triggers the Hexosamine Biosynthetic Pathway via the GCN2-ATF4 Signalling Pathway. Sci. Rep..

[268] Nabeebaccus A.A., Zoccarato A., Hafstad A.D., Santos C.X., Aasum E., Brewer A.C., Zhang M., Beretta M., Yin X., West J.A. (2017). Nox4 Reprograms Cardiac Substrate Metabolism via Protein O-GlcNAcylation to Enhance Stress Adaptation. JCI Insight.

[269] Jousse C., Oyadomari S., Novoa I., Lu P., Zhang Y., Harding H.P., Ron D. (2003). Inhibition of a Constitutive Translation Initiation Factor 2α Phosphatase, CReP, Promotes Survival of Stressed Cells. J. Cell Biol..

[270] Novoa I., Zeng H., Harding H.P., Ron D. (2001). Feedback Inhibition of the Unfolded Protein Response by GADD34-Mediated Dephosphorylation of EIF2α. J. Cell Biol..

[271] Wells L., Kreppel L.K., Comer F.I., Wadzinski B.E., Hart G.W. (2004). O-GlcNAc Transferase Is in a Functional Complex with Protein Phosphatase 1 Catalytic Subunits. J. Biol. Chem..

[272] Slawson C., Lakshmanan T., Knapp S., Hart G.W. (2008). A Mitotic GlcNAcylation/Phosphorylation Signaling Complex Alters the Posttranslational State of the Cytoskeletal Protein Vimentin. Mol. Biol. Cell.

[273] van Huizen R., Martindale J.L., Gorospe M., Holbrook N.J. (2003). P58IPK, a Novel Endoplasmic Reticulum Stress-Inducible Protein and Potential Negative Regulator of EIF2α Signaling*. J. Biol. Chem..

[274] Ray M.K., Datta B., Chakraborty A., Chattopadhyay A., Meza-Keuthen S., Gupta N.K. (1992). The Eukaryotic Initiation Factor 2-Associated 67-KDa Polypeptide (P67) Plays a Critical Role in Regulation of Protein Synthesis Initiation in Animal Cells. Proc. Natl. Acad. Sci. USA.

[275] Li X., Chang Y.H. (1996). Evidence That the Human Homologue of a Rat Initiation Factor-2 Associated Protein (P67) Is a Methionine Aminopeptidase. Biochem. Biophys. Res. Commun..

[276] Datta B., Ray M.K., Chakrabarti D., Wylie D.E., Gupta N.K. (1989). Glycosylation of Eukaryotic Peptide Chain Initiation Factor 2 (EIF-2)-Associated 67-KDa Polypeptide (P67) and Its Possible Role in the Inhibition of EIF-2 Kinase-Catalyzed Phosphorylation of the EIF-2 α-Subunit*. J. Biol. Chem..

[277] Datta B., Datta R., Mukherjee S., Zhang Z. (1999). Increased Phosphorylation of Eukaryotic Initiation Factor 2α at the G2/M Boundary in Human Osteosarcoma Cells Correlates with Deglycosylation of P67 and a Decreased Rate of Protein Synthesis. Exp. Cell Res..

[278] Datta R., Choudhury P., Ghosh A., Datta B. (2003). A Glycosylation Site, 60SGTS63, of P67 Is Required for Its Ability To Regulate the Phosphorylation and Activity of Eukaryotic Initiation Factor 2α. Biochemistry.

[279] Yu L., Chen Y., Tooze S.A. (2017). Autophagy Pathway: Cellular and Molecular Mechanisms. Autophagy.

[280] Hung Y.-H., Chen L.M.-W., Yang J.-Y., Yang W.Y. (2013). Spatiotemporally Controlled Induction of Autophagy-Mediated Lysosome Turnover. Nat. Commun..

[281] Papadopoulos C., Meyer H. (2017). Detection and Clearance of Damaged Lysosomes by the Endo-Lysosomal Damage Response and Lysophagy. Curr. Biol..

[282] Wyant G.A., Abu-Remaileh M., Wolfson R.L., Chen W.W., Freinkman E., Danai L.V., Heiden M.G.V., Sabatini D.M. (2017). MTORC1 Activator SLC38A9 Is Required to Efflux Essential Amino Acids from Lysosomes and Use Protein as a Nutrient. Cell.

[283] Mizushima N., Komatsu M. (2011). Autophagy: Renovation of Cells and Tissues. Cell.

[284] Kroemer G., Mariño G., Levine B. (2010). Autophagy and the Integrated Stress Response. Mol. Cell.

[285] He C., Klionsky D.J. (2009). Regulation Mechanisms and Signaling Pathways of Autophagy. Annu. Rev. Genet..

[286] Park S., Lee Y., Pak J.W., Kim H., Choi H., Kim J., Roth J., Cho J.W. (2015). O-GlcNAc Modification Is Essential for the Regulation of Autophagy in Drosophila Melanogaster. Cell Mol. Life Sci..

[287] Zachari M., Ganley I.G. (2017). The Mammalian ULK1 Complex and Autophagy Initiation. Essays Biochem..

[288] Audesse A.J., Dhakal S., Hassell L.-A., Gardell Z., Nemtsova Y., Webb A.E. (2019). FOXO3 Directly Regulates an Autophagy Network to Functionally Regulate Proteostasis in Adult Neural Stem Cells. PLoS Genet..

[289] Malta C.D., Cinque L., Settembre C. (2019). Transcriptional Regulation of Autophagy: Mechanisms and Diseases. Front. Cell Dev. Biol..

[290] Ho S.-R., Wang K., Whisenhunt T.R., Huang P., Zhu X., Kudlow J.E., Paterson A.J. (2010). O-GlcNAcylation Enhances FOXO4 Transcriptional Regulation in Response to Stress. FEBS Lett..

[291] Housley M.P., Udeshi N.D., Rodgers J.T., Shabanowitz J., Puigserver P., Hunt D.F., Hart G.W. (2009). A PGC-1alpha-O-GlcNAc Transferase Complex Regulates FoxO Transcription Factor Activity in Response to Glucose. J. Biol. Chem..

[292] Housley M.P., Rodgers J.T., Udeshi N.D., Kelly T.J., Shabanowitz J., Hunt D.F., Puigserver P., Hart G.W. (2008). O-GlcNAc Regulates FoxO Activation in Response to Glucose. J. Biol. Chem..

[293] Yoon I., Nam M., Kim H.K., Moon H.-S., Kim S., Jang J., Song J.A., Jeong S.J., Kim S.B., Cho S. (2020). Glucose-Dependent Control of Leucine Metabolism by Leucyl-TRNA Synthetase 1. Science.

[294] Kim K., Yoo H.C., Kim B.G., Kim S., Sung Y., Yoon I., Yu Y.C., Park S.J., Kim J.H., Myung K. (2022). O-GlcNAc Modification of Leucyl-TRNA Synthetase 1 Integrates Leucine and Glucose Availability to Regulate MTORC1 and the Metabolic Fate of Leucine. Nat. Commun..

[295] Jin L., Yuan F., Dai G., Yao Q., Xiang H., Wang L., Xue B., Shan Y., Liu X. (2020). Blockage of O-Linked GlcNAcylation Induces AMPK-Dependent Autophagy in Bladder Cancer Cells. Cell Mol. Biol. Lett..

[296] Shi Y., Yan S., Shao G.-C., Wang J., Jian Y.-P., Liu B., Yuan Y., Qin K., Nai S., Huang X. (2022). O-GlcNAcylation Stabilizes the Autophagy-Initiating Kinase ULK1 by Inhibiting Chaperone-Mediated Autophagy upon HPV Infection. J. Biol. Chem..

[297] Wang C., Wang H., Zhang D., Luo W., Liu R., Xu D., Diao L., Liao L., Liu Z. (2018). Phosphorylation of ULK1 Affects Autophagosome Fusion and Links Chaperone-Mediated Autophagy to Macroautophagy. Nat. Commun..

[298] Kaushik S., Cuervo A.M. (2018). The Coming of Age of Chaperone-Mediated Autophagy. Nat. Rev. Mol. Cell Biol..

[299] Zhang X., Wang L., Lak B., Li J., Jokitalo E., Wang Y. (2018). GRASP55 Senses Glucose Deprivation through O-GlcNAcylation to Promote Autophagosome-Lysosome Fusion. Dev. Cell.

[300] Dodson M., Liu P., Jiang T., Ambrose A.J., Luo G., de la Vega M.R., Cholanians A.B., Wong P.K., Chapman E., Zhang D.D. (2018). Increased O-GlcNAcylation of SNAP29 Drives Arsenic-Induced Autophagic Dysfunction. Mol. Cell Biol..

[301] D’Angelo G., Prencipe L., Iodice L., Beznoussenko G., Savarese M., Marra P., Tullio G.D., Martire G., Matteis M.A.D., Bonatti S. (2009). GRASP65 and GRASP55 Sequentially Promote the Transport of C-Terminal Valine-Bearing Cargos to and through the Golgi Complex*. J. Biol. Chem..

[302] Pothukuchi P., Agliarulo I., Pirozzi M., Rizzo R., Russo D., Turacchio G., Nüchel J., Yang J., Gehin C., Capolupo L. (2021). GRASP55 Regulates Intra-Golgi Localization of Glycosylation Enzymes to Control Glycosphingolipid Biosynthesis. EMBO J..

[303] Nüchel J., Tauber M., Nolte J.L., Mörgelin M., Türk C., Eckes B., Demetriades C., Plomann M. (2021). An MTORC1-GRASP55 Signaling Axis Controls Unconventional Secretion to Reshape the Extracellular Proteome upon Stress. Mol. Cell.

[304] Sou Y., Waguri S., Iwata J., Ueno T., Fujimura T., Hara T., Sawada N., Yamada A., Mizushima N., Uchiyama Y. (2008). The Atg8 Conjugation System Is Indispensable for Proper Development of Autophagic Isolation Membranes in Mice. Mol. Biol. Cell.

[305] Kabeya Y., Mizushima N., Ueno T., Yamamoto A., Kirisako T., Noda T., Kominami E., Ohsumi Y., Yoshimori T. (2000). LC3, a Mammalian Homologue of Yeast Apg8p, Is Localized in Autophagosome Membranes after Processing. EMBO J..

[306] Fracchiolla D., Sawa-Makarska J., Zens B., de Ruiter A., Zaffagnini G., Brezovich A., Romanov J., Runggatscher K., Kraft C., Zagrovic B. (2016). Mechanism of Cargo-Directed Atg8 Conjugation during Selective Autophagy. eLife.

[307] Jo Y.K., Park N.Y., Park S.J., Kim B.-G., Shin J.H., Jo D.S., Bae D.-J., Suh Y.-A., Chang J.H., Lee E.K. (2016). O-GlcNAcylation of ATG4B Positively Regulates Autophagy by Increasing Its Hydroxylase Activity. Oncotarget.

[308] Shaw J., Yurkova N., Zhang T., Gang H., Aguilar F., Weidman D., Scramstad C., Weisman H., Kirshenbaum L.A. (2008). Antagonism of E2F-1 Regulated Bnip3 Transcription by NF-ΚB Is Essential for Basal Cell Survival. Proc. Natl. Acad. Sci. USA.

[309] Hamacher-Brady A., Brady N.R., Logue S.E., Sayen M.R., Jinno M., Kirshenbaum L.A., Gottlieb R.A., Gustafsson Å.B. (2007). Response to Myocardial Ischemia/Reperfusion Injury Involves Bnip3 and Autophagy. Cell Death Differ..

[310] Tracy K., Dibling B.C., Spike B.T., Knabb J.R., Schumacker P., Macleod K.F. (2007). BNIP3 Is an RB/E2F Target Gene Required for Hypoxia-Induced Autophagy. Mol. Cell Biol..

[311] Rao X., Duan X., Mao W., Li X., Li Z., Li Q., Zheng Z., Xu H., Chen M., Wang P.G. (2015). O-GlcNAcylation of G6PD Promotes the Pentose Phosphate Pathway and Tumor Growth. Nat. Commun..

[312] Yi W., Clark P.M., Mason D.E., Keenan M.C., Hill C., Goddard W.A., Peters E.C., Driggers E.M., Hsieh-Wilson L.C. (2012). Phosphofructokinase 1 Glycosylation Regulates Cell Growth and Metabolism. Science.

[313] Mullarky E., Cantley L.C. (2015). Diverting Glycolysis to Combat Oxidative Stress. Innovative Medicine.

[314] Wang Y., Liu J., Jin X., Zhang D., Li D., Hao F., Feng Y., Gu S., Meng F., Tian M. (2017). O-GlcNAcylation Destabilizes the Active Tetrameric PKM2 to Promote the Warburg Effect. Proc. Natl. Acad. Sci. USA.

[315] Nie H., Ju H., Fan J., Shi X., Cheng Y., Cang X., Zheng Z., Duan X., Yi W. (2020). O-GlcNAcylation of PGK1 Coordinates Glycolysis and TCA Cycle to Promote Tumor Growth. Nat. Commun..

[316] Burt R.A., Dejanovic B., Peckham H.J., Lee K.A., Li X., Ounadjela J.R., Rao A., Malaker S.A., Carr S.A., Myers S.A. (2021). Novel Antibodies for the Simple and Efficient Enrichment of Native O-GlcNAc Modified Peptides. Mol. Cell Proteom..

[317] Gorelik A., Bartual S.G., Borodkin V.S., Varghese J., Ferenbach A.T., van Aalten D.M.F. (2019). Genetic Recoding to Dissect the Roles of Site-Specific Protein O-GlcNAcylation. Nat. Struct. Mol. Biol..

[318] Shan H., Sun J., Shi M., Liu X., Shi Z., Yu W., Gu Y. (2018). Generation and Characterization of a Site-Specific Antibody for SIRT1 O-GlcNAcylated at Serine 549. Glycobiology.

